# Afghan Hindu Kush: Where Eurasian Sub-Continent Gene Flows Converge

**DOI:** 10.1371/journal.pone.0076748

**Published:** 2013-10-18

**Authors:** Julie Di Cristofaro, Erwan Pennarun, Stéphane Mazières, Natalie M. Myres, Alice A. Lin, Shah Aga Temori, Mait Metspalu, Ene Metspalu, Michael Witzel, Roy J. King, Peter A. Underhill, Richard Villems, Jacques Chiaroni

**Affiliations:** 1 Aix Marseille Université, ADES UMR7268, CNRS, EFS-AM, Marseille, France; 2 Estonian Biocentre and Department of Evolutionary Biology, University of Tartu, Tartu, Estonia; 3 Sorenson Molecular Genealogy Foundation, Salt Lake City, Utah, United States of America; 4 Department of Psychiatry, Stanford University School of Medicine, Stanford, California, United States of America; 5 Department of Biochemistry, Kabul Medical University, Kabul, Afghanistan; 6 Department of South Asian Studies, Harvard University. Cambridge, Massachusetts, United States of America; 7 Department of Genetics, Stanford University School of Medicine, Stanford, California, United States of America; 8 Estonian Academy of Sciences, Tallinn, Estonia; Erasmus University Medical Center, The Netherlands

## Abstract

Despite being located at the crossroads of Asia, genetics of the Afghanistan populations have been largely overlooked. It is currently inhabited by five major ethnic populations: Pashtun, Tajik, Hazara, Uzbek and Turkmen. Here we present autosomal from a subset of our samples, mitochondrial and Y- chromosome data from over 500 Afghan samples among these 5 ethnic groups. This Afghan data was supplemented with the same Y-chromosome analyses of samples from Iran, Kyrgyzstan, Mongolia and updated Pakistani samples (HGDP-CEPH). The data presented here was integrated into existing knowledge of pan-Eurasian genetic diversity. The pattern of genetic variation, revealed by structure-like and Principal Component analyses and Analysis of Molecular Variance indicates that the people of Afghanistan are made up of a mosaic of components representing various geographic regions of Eurasian ancestry. The absence of a major Central Asian-specific component indicates that the Hindu Kush, like the gene pool of Central Asian populations in general, is a confluence of gene flows rather than a source of distinctly autochthonous populations that have arisen in situ: a conclusion that is reinforced by the phylogeography of both haploid loci.

## Introduction

The Hindu Kush covers the mountainous regions of Afghanistan and north Pakistan, including areas on the western borders of the Pamir Mountains; since ancient times it has been the crossroad of the more densely settled regions of South and Central Asia and of historical Persia. The Hindu Kush mountains have forests above 800–1000 meters and alpine meadows below; several old Iranian texts, such as the Avesta, refer to this territory as being rich in vegetal resources [1]. This made the Hindu Kush a favored area for transhumance, as well as a pathway from the Ural steppe area, bypassing the West Central Asian deserts, towards Afghanistan and Eastern Iran, in addition to following the paths of Central Asian rivers [2].

The earliest archaeological evidence of modern humans in the area dates back some 30,000 years; it was found in the northwest of Pakistan on the South Asian side of the Hindu Kush [3]. The archaeological and linguistic data from the Bronze Age era present sequences in time and space relevant to prehistoric settlement in the Hindu Kush. Urban culture flourished in the region, beginning with the widespread BMAC (Bactria-Margiana Archaeological Complex) of Afghanistan and Turkmenistan, late in the third millennium BC [2], [4], [5]. The unknown BMAC language can be triangulated from the loan words that it transmitted to Old Iranian (Avestan, Old Persian), Old Indian (Vedic) and Tocharian; the latter was spoken in westernmost China (Xinjiang) [6]–[9]. This language seems related to North Caucasian in the west and to Burushaski from the high Pamirs in the east, both form part of the Macro-Caucasian language family that also includes Basque [10], [11].

Later historical and linguistic evidence points to the Hindu Kush as being a region reached by the early expansion of the Indo-Iranian languages [12], [13]. They covered the earlier BMAC level, expanding from the northern steppe (Andronovo culture) after 2000 BC [14]–[16], possibly through the Inner Asian Mountain Corridor pathway that stretched from the northern steppe belt to the Hindu Kush [2]. By 1400 BC the Indo-Aryan branch of Indo-Iranian languages covered the western part of Central Asia from the Urals to the Hindu Kush and the eastern borders of Mesopotamia [17].

After circa 1000 BC this extensive Indo-Aryan layer was in turn overlapped by their close relatives, the Iranians. They practiced horseback nomadism across Asia, from the borders of Rumania to Xinjiang (Scythians, Saka) with some of them also settling in the Hindu Kush (Bactrians), the Tien Shan area (Sogdians), and as far west as present-day Iran (Medes, Parthians, Persians) [12], [13]. In this large Iranian speaking area, people could easily move both east and west along the steppe belt, helped in travel, herding and warfare by the development of horseback riding [18].

Pastoral nomadism in western Central Asia, and in parts of eastern Central Asia, was characterized by Indo-European speakers first, followed by Indo-Iranian, then Iranian, until Turkic, Altaic-speaking people finally took over. The Kalash from Chitral in northwestern Pakistan provide an isolated illustration of such movements: there is no evidence of admixture between them and East Asians [19], and they preserve, even today, many traces of early Indo-Aryan (pre-Vedic) mythology and rituals, while their language corresponds to very archaic Indo-Aryan [20], [21].

History shows commerce and conquests meandering through the Hindu Kush region. Alexander the Great's army subdued the area around 330 BC [22]. During the Greco-Roman and early medieval periods, the Hindu Kush became an active way station for trade along the Silk Road, which connected the Mediterranean Basin and Eastern Asia for over 16 centuries [23], [24].

Around 600 AD, the western part of central Asia was invaded by nomad Turkic tribes who established the currently Turkic speaking areas [25]. These tribes replaced the former Iranian-speaking populations, though small enclaves still remained in North Uzbekistan in 1400 AD (Khwarezmian), as they do even today in the western and southern valleys of the Pamir Mountains, as well as near Samarkand in Uzbekistan (Yaghnobi) and in southwestern Xinjiang (Sariqoli) [12], [25], [26].

The presence of nomad Turkic tribes was first reported around 200 BC with the creation of the first Central Asian nomad empire. The Turkic conquests went on for a thousand years when they were interrupted by the Mongol expansion after 1200 AD: Genghis Khan's vast empire stretched from the lower Danube to the Pacific, including much of Siberia, northern-central China and the Il-Khanate' that covered the Anatolian and Persian areas south of the Black and Caspian Seas and of the Hindu Kush [25], [27], [28].

Several late migrations took place simultaneously around 1000 AD: the western Iranian-speaking Baluchis moved eastward from eastern Turkey into Baluchistan, the Dravidian-speaking Brahui migrated north from Central India, and the Romani (Gypsies) migrated westward out of India [29].

Despite this rich and complex history, the few genetic studies devoted to Afghanistan have been restricted to Y-chromosome and/or to the Pashtun population [30]–[32].

Although the historic Mongol incursions have been strongly supported by genetic studies [33]–[35], interpretation of genetic data concerning prehistoric events in Central Asia is still controversial. A publication based on Y-chromosome data proposed that Central Asia was a source of at least 3 major waves of migration leading into Europe, the Americas and India [36].

However, mitochrondrial DNA (mtDNA) haplogroup diversity in populations living in Turkey, Georgia, Iran, and Central Asia suggested that the predominant direction of gene flow was from west (the Fertile Crescent) to east (Pakistan) [37]–[42]. This alternative hypothesis is further supported by a recent genome wide (GWAs) study [43] consistent with such a western influx during the Neolithic period, involving linguistic changes, caprine domestication, and wheat farming [1], [6], [44]–[46]. Such results seem to be consistent with the linguistic and ethnic changes described above.

Whether Central Asia was a source or a convergent zone of sub-continent gene flows remains unresolved. To address this issue, we present mt-DNA and Y-chromosome data on more than 500 Afghan samples from 5 main ethnic groups inhabiting the mountainous region of the Hindu Kush: Tajik, Turkmen, Pashtun, Hazara and Uzbek. Mt-DNA analysis consisted in HV1 sequence and polymorphism analysis in the coding region. Y-chromosome analysis covered 102 binary SNPs, among which there were 6 new markers, and 39 Y-STRs. A representative subset of each ethnic group was also analyzed for autosomal markers by Illumina 650 K SNP. This Afghan data was completed with the same Y-chromosome analyses of 672 original male samples from Iran, Kyrgyzstan, Mongolia and updated Pakistani samples (HGDP-CEPH). Finally, the results were compared to databases built up from published literature for the purpose of the present study using autosomal results from 1183 individuals; 14,308 HV1 sequences concerning mt-DNA analysis and results from 34 harmonized Y haplogroups including 8,111 individuals.

## Materials and Methods

### Sampling

**Figure 1:**
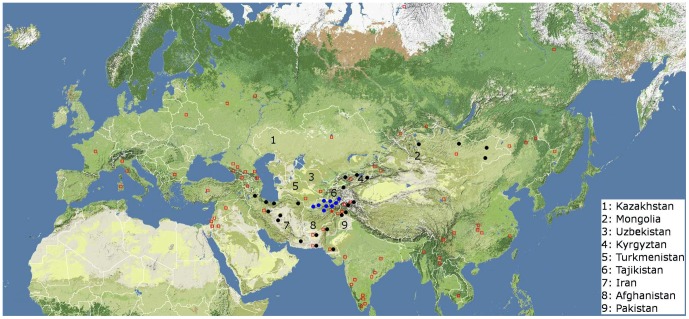
Samples collection locations. Blue dots indicate locations where samples were collected in Afghanistan and analyzed for mt DNA, Y-chromosome and GWA, red dot indicates Afghan capital, Kabul. Black dots indicate locations where samples were collected in Iran, Kyrgyzstan, Mongolia and Pakistan (HGDP-CEPH) and analyzed for Y-chromosome (see further description in Table S1). Red squares indicate samples locations used for the autosomal analyses (PCA, Fst, structure-like ADMIXTURE) (see further description in Table S2).

A total of 516 samples of blood obtained by venipuncture were collected from 5 ethnically distinct populations in the Hindu Kush region of Afghanistan: Hazara, Tajik, Uzbek, Turkmen, and Pashtun (Figure 1 and Table S1). 478 additional original samples were also collected for Y- chromosome analysis from Iran, Kyrgyzstan and Mongolia as well as 177 updated Pakistani samples (HGDP-CEPH) [47] (Figure 1 and Table S1).

All samples were obtained from unrelated male volunteers after approval of the informed consent protocols. The study protocol was registered by the *Ministère de l'Enseignement Supérieur et de la Recherche* in France (committee 208C06, decision AC-2008-232).

### DNA extraction

DNA was purified from blood using the Qiamp blood kit (Qiagen, Courtaboeuf, France) and DNA concentrations were determined by spectrometry.

### Autosomal genetic analyses

Autosomal genetic variation was analyzed in a subset of 5 Hazara, 5 Tajik, 5 Uzbek, 4 Turkmen and 5 Pashtun samples using Illumina 650 K SNP array. In addition 1485 samples were taken from published data [43], [48]–[53] (See Figure 1 and Table S2 for population description from published data). Admixture analyses were performed including African populations given the African component in some populations in Pakistan (Makrani, Balochi, Brahui). No Native American populations were included in the Admixture analyses. Autosomal genotypic data may be accessed through The National Center for Biotechnology Information-Gene Expression Omnibus (NCBI GEO), by request to the authors or on the website http://evolutsioon.ebc.ee/MAIT/public_data/index.html.

*Quality control*

We used PLINK 1.07 [54] in order to only retain SNPs on the 22 autosomal chromosomes that had a minor allele frequency >1%, a genotyping success >97%; additionally, only individuals that had a genotyping success rate >97% were used. Also, since Linkage Disequilibrium (LD) can affect principal component and structure-like analyses, the marker set was further thinned by excluding SNPs in strong LD (pairwise genotypic correlation r^2^>0.4) in a window of 200 SNPs (sliding window of 25 SNPs) [52]. On average, approximately 232,000 SNPs were left depending on the populations/samples retained for the specific analyses.

#### Genetic clustering analysis

To study the population's genetic structure in the dataset of over 232,000 genome-wide SNPs, we used a structure-like [55] model-based maximum likelihood (ML) clustering approach implemented in ADMIXTURE [56]. For a given number (K) of constructed ancestral populations, the algorithm assigns to each individual ancestry fractionsin each of the K constructed ancestral populations. To ensure the ancestry assignments, ADMIXTURE was run 100 times for each K (K = 2 through K = 15, Figure S1). Best runs are defined by the highest Loglikelihood scores (LLs) coupled with minimal differences between LLs, that is <1. As seen from the 10% top fraction of the K = 2 to K = 15 runs, these conditions were met from K = 2 to K = 11, and thus K = 2-to-11 were assumed to have reached the global maximum of the inference. The best K as ascertained by the lowest cross-validation indexes was K = 9 [56].

#### Frequency map

A color is assigned to each K. Each individual is partitioned into K colored components, which represent the individual's estimated fractions of shared genetic background, or ancestry component (AC). In order to depict the spatial distribution of the ACs detected in Eurasia, the proportions of ACs 3, 4, 6, 7, 8 and 9 as resolved at K = 9 were then displayed on a color-graded map.

#### Geographic correlation

Correlation between spatial distribution of the ACs detected in Eurasia and each proportion of ACs 1 to 9 as resolved at K = 9 was tested with the Pearson test (significance alpha value = 0.05) using XLSTAT 7.5.2 software.

#### Principal component analysis and F_ST_

As the inclusion of African samples results in the first Principal Component (PC) sorting African samples versus non-African samples, the former were excluded from the analysis. The LD pruning procedure was repeated on the 1183 samples left. Pairwise genetic differentiation between populations with sample size >5 was estimated with the F_ST_ index. PCA and *F*_ST_ calculations were performed using the SmartPCA program [57].

### Haploid genetic analyses

Mitochondrial DNA was analyzed from 90 Pashtun, 146 Tajik, 78 Hazara, 75 Turkmen and 127 Uzbek. Samples were sequenced between nucleotide positions 15900 and 16569. Further analyses were performed by RFLP and/or direct sequencing of polymorphisms of the coding region at 29 nucleotide positions: 1406, 1438, 3010, 3816, 3915, 3992, 4188, 4336, 4580, 4769, 4793, 7735, 7789, 8598, 8818, 10217, 10556, 11696, 12007, 13167, 14178, 14182, 14582, 14668, 14872, 15259, 15908, 15940, and 15968. The mutations were scored compared to RSRS [58] and haplogroup affiliation was defined according to the latest build of Phylotree.org at the time (build 11, February 7^th^) [59].

Eighty seven Pashtun, 142 Tajik, 77 Hazara, 74 Turkmen and 127 Uzbek obtained successful Y-chromosome analysis. In order to analyze a representative geographical coverage at the same level of resolution, additional populations also had Y-chromosome analysis: 9 populations from Iran totalizing 186 individuals, 6 populations from Kyrgyzstan totalizing 150 men including Dungan and Uygur individuals, 4 populations from Mongolia totalizing 160 samples and the eight Pakistani populations from the HGDP-CEPH DNA collection [47], [60] totalizing 176 successfully typed individuals (Table S1 and Figure 1). These samples were phylogenetically resolved in a hierarchical manner for 102 binary markers including 6 new markers (Table S3). These new Y markers were discovered independently in Dr. Underhill's laboratory using DHPLC methodology as part of his ongoing search for polymorphisms in all human Y-chromosome haplogroups. Five of these new markers belong to haplogroup C3 (M386-C3a, M532-C3b, M504-C3b2b, M546-C3b2b, M401-C3b2b1); this haplogroup is characteristic of Mongol expansion and has been described in Hazara [61].

Following PCR amplification, binary marker genotyping was accomplished by either Denaturing High Performance Liquid Chromatography (DHPLC), RFLP analysis, Taqman® (Applied Biosystems) assay or direct sequencing methodology. Nomenclature assignments were defined according to the International Society of Genetic Genealogy Haplotype 2012 Tree [62] that provides a catalogue of current refinements.

Additionally, a total of 39 Y-STRS (DYS385a-b, DYS388, DYS389a, DYS389B, DYS390, DYS391, DYS392, DYS393, DYS394/19, DYS426, DYS437, DYS438, DYS439, DYS441, DYS442, DYS444, DYS445, DYS446, DYS447, DYS448, DYS449, DYS452, DYS454, DYS455, DYS456, DYS458, DYS459a-b, DYS460, DYS461n(TAGA)n, DYS462, DYS463, GGAAT1B07, YCAIIa-b, YGATAA10, YGATAC4/Y_DYS635, YGATAH4) were genotyped using two multiplex reactions. Electrophoresis of the amplified fragments, mixed with formamide and 500 LIZ internal Size Standard was carried out in an ABIPRISM 31030XL Genetic Analyzer. Interpretation was performed by GeneScan ID 3.2 fragment analysis software.

### Haploid database construction

We gathered mtDNA and Y-chromosome haplogroup frequency data from published data focused on Central Asian populations.

Concerning mtDNA, a total of 14,308 HV1 sequences from 214 populations were included (Table S4). Haplogroups were assigned according to Phylotree.org (build 11, February 7^th^) [59].

Concerning the Y-chromosome, the data set was initially built from 442 Eurasian populations totalizing 23,800 men from 68 bibliographic references. Since the studies have not all used the same level of resolution for SNP genotype samples, we needed to determine the consensus level of phylogenetic depth in the Y-chromosome tree. We therefore agreed to 34 male lineages and summed all frequencies within each: C-M130(xPK2), C3a-PK2, D-M174, E-M96(xP147xM75), E1-P147, E2-M75, F-M89, G-M201, G1-M285, G2-P287, H-M69, H1a-M82, K-M9, I-M258, I1-M253, I2-M438, J-M304, J1-M267, J2-M172, J2a-M410, L-M11, L1a-M76, L1b-M317, M-P256, N-M231, O-M175, P-M74, Q-M242 (xM25), Q1a2-M25, R-M207 (xM449, M343, M479), R1a-M449, R1b-M343, R2-M479, and T-M70. Then sample sizes of less than 10 individuals were eliminated. The final Y-chromosome data set encompassed 8,111 individuals from 187 populations (Table S4).

### Haploid statistical analyses

#### Distinctive haplogroups

For both haploid markers, we identified the most discriminative lineages. We estimated the chi-square values based on haplogroup frequencies and selected the haplogroups with significant (p<0.05) differences of frequencies between at least one couple of populations [63].

#### Y-Chromosome genetic diversity

Y-Chromosome haplotype and haplogroup diversities were calculated for each population with the ARLEQUIN v3.5.1.2 package [64]. Correlation between haplogroup diversity and haplotype diversity for was calculated using the Pearson test with GRAPH PAD Prism 5.

#### Analysis of Molecular Variance (AMOVA)

For both of the haploid markers, gene diversity indexes and AMOVA were performed with the ARLEQUIN v3.5.1.2 package [64]. The Fct value, described as the diversity among groups of populations, was used to estimate genetic structure.

Concerning the Y-chromosome, we used the 37 populations from Afghanistan, Iran, Kyrgyzstan, Mongolia and Pakistan screened for the high-resolution 102 Y-SNPs. In order to fairly compare the genetic structure of the female population with that of the male one, we selected a subset of 27 populations from Iran, Mongolia and Kyrgyzstan (totalizing 3067 HVS-I sequences) from the mtDNA database described above (Table S4) and compared them with our Afghan data.

#### Factorial Correspondence Analysis

We ran two levels of factorial correspondence analysis (FCA) using XLSTAT 7.5.2 software. Given the depth of resolution of the 102 Y-SNPs herein examined, we first focused on the genetic relationships between the 84 most-derived male lineages in 37 populations from Afghanistan, Iran, Pakistan, Kyrgyzstan and Mongolia. Afterwards, we extended the comparison between populations to a sub-continental scale using our databases described above (Tables S3).

#### Median Joining Network

Networks were constructed by the median joining method using Network 4.5.0.2, where ε = 0 and microsatellite loci were weighted proportionally to the inverse of the repeat variance observed in each haplogroup [65].

#### Spatial correlation of Y-chromosome data

To further explore the links between Y-chromosome distribution and geography, we first conducted a Mantel test using 37×37 matrices of Fst and geographic distance (in kilometers using version 1.2.3 of the Geographic Distance Matrix Generator). To elucidate the nature of this association, we investigated the role of latitude and longitude on the frequency distribution of the major Eurasian Y haplogroup, suggested as genetic markers of the most striking historical events (Mongol expansion, Neolithic demic diffusion, Indo-Iranian expansion): C3b2b1-M401, J2a1-Page55 and R1a1a-M198 [35], [46], [66].

## Results

### Autosomal analyses

**Figure 2:**
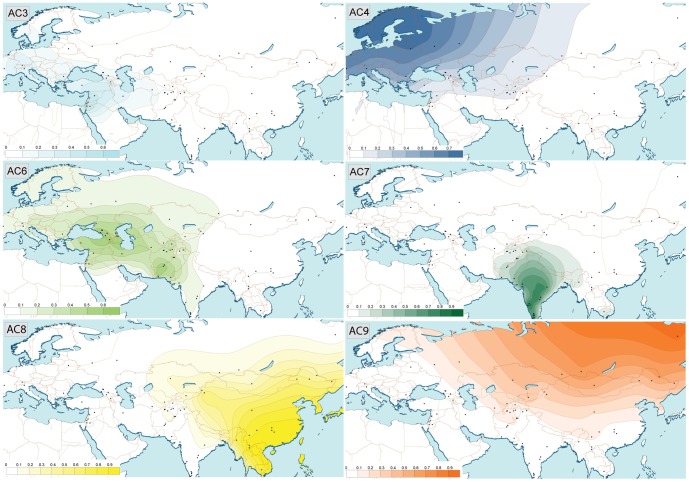
Spatial distribution of Ancestry Components based on the admixture analysis results at K = 9. Frequency data (ancestry fractions) were converted by applying the Kriging algorithm using the software Surfer v8.00. The color for the respective ACs matches that of Figures S1 and S2.

Autosomal variation in Eurasian populations was analyzed via genetic structure in a dataset of over 232,000 genome-wide SNPs, depicted by a structure-like clustering approach implemented in ADMIXTURE. None of the genetic structure simulations (K = 2 to K = 15, see Figure S1) show any ancestral component (AC) specific to, or even dominant in Central Asia, except for the Kalash (see below). We identified nine ACs which reflect geographically localized sets of SNPs with shared genetic ancestry in these regions. To get a better idea of the spatial distribution of the so-defined autosomal ACs, the proportions of AC 3, 4, 6, 7, 8 and 9 as resolved at K = 9 (Figure S2) were depicted on a map (Figure 2). The proportions of AC 3, 4, 6, 7, 8 and 9 as resolved at K = 9 displayed high correlation with geography, either with latitude or with longitude, or both (Figure S3). AC3 which is dominant in Middle Eastern populations has its highest frequency in Lebanon/Sinai, is present westward in Europe until the Atlantic Ocean and gradually decreases eastwards until the western part of Afghanistan; AC3 is correlated with longitude. AC4 has its highest frequency in north-west of Europe and decreases in the south until the northern and eastern coasts of the Mediterranean and eastwards until the northern half of Afghanistan; AC4 is correlated both with longitude and latitude. In the case of the light green AC 6, there are two peaks of moderately high frequency, one in the Caucasus, the other in the Indus Basin; Afghanistan lies between these spots. This AC covers all Western Europe, the western part of Russia, the extreme west of China and half of India. AC6 is correlated with longitude. AC7 is high in the extreme south of India and decreases northwards until the borders of Pakistan, Afghanistan and the south western part of China. AC7 is correlated with latitude. AC8 displays its highest frequency in South East Asia and decreases westwards until reaching Afghanistan and Kazakhstan; AC8 is correlated with longitude. AC9 displays its highest frequency in the extreme north east of Russia and decreases southwards and westwards until reaching Scandinavia, the western border of Russia, Turkmenistan, Afghanistan, the northern border of India and the northern half of China. AC9 is correlated both with longitude and latitude. The general pattern observed is a rather distinct sub-continental partition, with one geographical peak of frequency and a gradual decline of frequency either side of it. This picture obtained with autosomal data is strikingly similar to the ones described with mtDNA [67] or the Y-chromosome [68], [69]. Overall, none of these subcontinental ACs revolve around Central Asia but decline towards it instead.

The Afghan Hindu Kush samples, in line with other Central Asian populations (see Table S2), are characterized by a mixture of ACs that are dominant in East, South or West Eurasia. Notably, at K = 9, all AC, except AC1, 2 and 5, reach Afghanistan with various degrees of frequency and could be detected in the examined genomes (Figure S2). Although the respective proportions of East Asian and Siberian ACs (8 and 9) are particularly high among the Turkic speakers of Central Asia, they are not always correlated to Turkic languages, as exemplified by the Turkmen population. Indeed, even among Indo-European speakers, the ACs 8 and 9 can reach rather high proportions; although it is not surprising in the case of Afghan and Pakistani Hazara who are both known to derive from Mongol populations [70]–[72], such patterns are noteworthy for Pashtun and Tadjik populations. It should be pointed out that the Kalash differ from this analysis. At K = 7, they exhibit two main ACs, one being predominant in Europe and the Caucasus (dark blue AC 4) and the other in the Indus Basin and the Indian sub-continent (dark green AC 5). At K = 9, the Kalash acquire their own specific AC reflecting doubtlessly restricted gene flows into this long-term remote ethnic group [19], [49].

Our autosomal data, plotted as a colored heat map of Fst distances (Figure S4) further confirm the genetic patterns previously described by Yunusbayev et al. [53] and reveal Central Asia as being quite homogeneous despite its linguistic heterogeneity. Notably, the 5 Afghan groups under study display little genetic distance between pairs. In this cluster, Turkmen from Turkmenistan, Kazakh and Kyrgyz populations are more distant genetically; and the Altaic-Turkic-speaking Uzbek from Uzbeskistan, Kazakh, Kyrgyz and Uyghur populations show the smallest genetic distances with the Siberian and East Asian populations.

**Figure 3:**
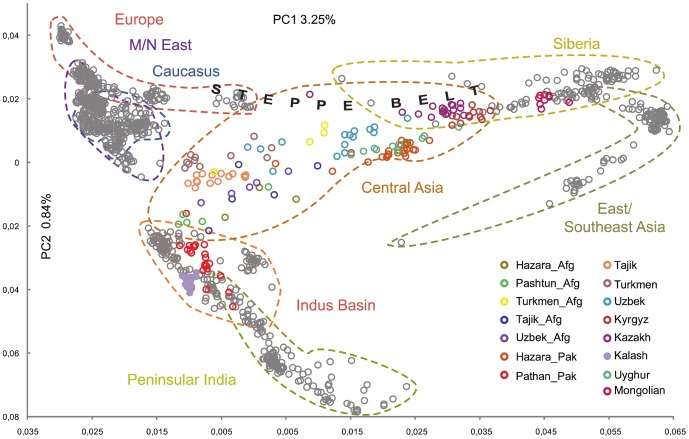
First and second components of the Principal Component Analysis based on autosomal data. The corresponding colored dots for the Central Asian populations are shown on the lower right corner. The colored “arrows” on the background represent the frequency gradients as seen as on Figures S1 and S2 and follow the same color code. It shall be stressed that they DO NOT represent actual gene flow, PCA analysis does not permit to reveal such movements. _Pak and _Afg stand for Pakistan and Afghanistan respectively.

The sub-continent clustering is apparent in the Principal Component Analysis (PCA) (Figure 3). The first Principal Component separates Western Eurasia (including the Indian sub-continent) from Eastern Eurasia reflecting a west/east axis, with Central Asia marking the transition zone. The second PC separates the Indian sub-continent from Eurasia. Among the broad geographic regions, Europe, the Middle and Near East, Caucasus and the Indus Basin display the tightest clusters; Peninsular India, Siberia and East/South Asia clusters are rather broad; whereas the Central Asia cluster is the most diffuse and loose, sitting at the convergence of the axes described above. The Altaic speaking populations appear in different parts of this cluster whereas the Indo-European speaking populations lie in the left part, with the exception of the Hazara. Interestingly, while the Pakistani Hazara form a tight cluster, the Hazara in the Afghan Hindu Kush are more spread out. Moreover, Tajik, Uzbek and Turkmen samples collected in Afghanistan do not genetically behave like those in their respective eponymous republics. On the contrary, the Pashtun, whether from Afghanistan or Pakistan, form a more genetically homogeneous ethnic group.

### Mitochondrial DNA

#### Diversification

Using haplogroup frequencies (Figure S5), we focused on discriminant haplogroups that could help describe the genetic relationship between the 5 Afghan ethnic groups under study. Because of the very large diversity of mitochondrial haplogroups described here, they were gathered into the following 14 main haplogroups: C4, F1, Z3, Z7, R0, T, U5, W3, J1, U7, M30, M4, U2 and R2. We observed a close pattern between Tajik and Uzbek. Their only differences are the absence of haplogroup F1 and a very low frequency of U5 in Uzbek (p<0.01), whereas, Tajik lack both M4 (p<0.02) and Z3 haplogroups. The Turkmen population is characterized by the complete absence of the U5 and U7 haplogroups that are present in all other populations (p<0.03). The Pashtun population is characterized by a high frequency of U2 (p<0.05) and R0 haplogroups and the exclusive presence of haplogroup Z7 (p<0.05). Furthermore, Pashtun are the only population to lack M30 (p<0.01), W3 (p<0.04) and Z3 haplogroups. Concerning the Hazara population, they show the highest frequencies for F1 (p<0.01), C4 (p<0.02), M30 (p<0.02) and Z3 (p<0.05) haplogroups. In addition, the Hazara lack J1 and T haplogroups, present in all other Hindu Kush populations studied (p<0.05). Although the Hazara population has the highest percentage of haplogroups typical of East Eurasia (33.3%), the lower level of resolution of published data does not allow to trace them to specific populations.

#### Factorial Correspondence Analysis

First and second axes of the Factorial Correspondence Analysis are represented in Figure S6. First and second axes account respectively for 13.27% and 10.70% of the total variance. Axis1 is mainly driven by East Eurasian (such as C, D, F, G) and South Asian haplogroups (macrohaplogroups M and U2). The second PC is driven by East and West Eurasian haplogroups. The general overview offers a triangular distribution of the populations; linguistic and geographical assignations have been highlighted.

Figure S6-A shows the populations colored according to their linguistic affiliation. Axis 1 differentiates the Altaic from Dravidian and Indo-European speakers, while the Caucasian speakers stand at the meeting point. Axis 2 separates the Caucasian from the Sino-Tibetan, Dravidian and most of the Altaic Indo-European speakers. In detail each linguistic phylum displays a specific distribution (Figures S6-B and C). Among Altaic speakers, Tungusic speakers are grouped on the edge of the Altaic cluster, the Mongolic speakers also form a tight cluster which partially overlaps the Tungunsic cluster and the Turkic cluster. The Turkic speakers are the most dispersed, overlapping clusters respectively made up of Tungusic, Mongolic, Caucasian and Indo-European (namely Indo-Iranian) clusters. Concerning the Indo-European phylum, Slavic, Armenian and Iranian branches are split from Indo-Aryan according to axis 1. Notably, Indo-Aryan clusters with Dravidian speakers. When we consider our Afghan samples, they show central positions; Tajik, Uzbek and Turkmen populations are closer to Indo-Iranian and Caucasus clusters, Pashtun are close to the Indo-Aryan cluster, and Hazara are, as expected, near to the Altaic cluster. Figure S6-D shows the population colored according to main geographic regions. While Central Asian populations do not cluster, the three points of the general triangular distribution formerly observed are i) South Asia, ii) East Asia and Siberia and iii) Caucasus and West Asia.

#### AMOVA

The intergroup variance between the Hindu Kush populations and data from published literature ranges from 1.29% when sorted according to language (Indo-European and Altaic, p<0.01) to 1.76% when sorted according to geography (Afghanistan, Mongolia and Kyrgyzstan, p<0.001).

We then tested numerous combinations of population clustering to deduce the best population structure based on our observations from the autosomal PCA (Figure 3) and haplogroup frequency distributions. The two highest Fct are obtained when Mongol and Kyrgyz populations form a separate core from Pashtun, Tajik, Uzbek and Turkmen populations (Fct = 2.22% and 2.08% respectively, both p<0.001). Interestingly, Hazara do not change the population structure when associated with Northeastern populations (Mongol and Kyrgyz) or associated with the Afghan populations (Pashtun, Tajik, Uzbek and Turkmen).

### Y-Chromosome

#### Diversification

Phylogenetic relationships, haplogroup frequencies and haplogroup and haplotype diversities are presented in Figure S7. Y-Chromosome STR data of each individual are presented in table S5. 94% of the chromosomes are distributed within the following 9 main haplogroups: R-M207 (34%), J-M304 (16%), C-M130 (15%), L-M20 (6%), G-M201 (6%), Q-M242 (6%), N-M231 (4%), O-M175 (4%) and E-M96 (3%). Within the core haplogroups observed in the Afghan populations, there are sub-haplogroups that provide more refined insights into the underlying structure of the Y-chromosome gene pool. One of the important sub-haplogroups includes the C3b2b1-M401 lineage that is amplified in Hazara, Kyrgyz and Mongol populations. Haplogroup G2c-M377 reaches 14.7% in Pashtun, consistent with previous results [31], whereas it is virtually absent from all other populations. J2a1-Page55 is found in 23% of Iranians, 13% of the Hazara from the Hindu Kush, 11% of the Tajik and Uzbek from the Hindu Kush, 10% of Pakistanis, 4% of the Turkmen from the Hindu Kush, 3% of the Pashtun and 2% of the Kyrgyz and Mongol populations. Concerning haplogroup L, L1c-M357 is significantly higher in Burusho and Kalash (15% and 25%) than in other populations. L1a-M76 is most frequent in Balochi (20%), and is found at lower levels in Kyrgyz, Pashtun, Tajik, Uzbek and Turkmen populations. Q1a2-M25 lineage is characteristic of Turkmen (31%), significantly higher than all other populations. Haplogroup R1a1a-M198/M17 is characterized by its absence or very low frequency in Iranian, Mongol and Hazara populations and its high frequency in Pashtun and Kyrgyz populations.

Kyrgyz and Pashtun display the lowest Y-chromosome genetic diversity, whereas populations from Iran show the highest Y-chromosome genetic diversity (Figure S8-A). Haplogroup and haplotype diversities are highly correlated (Figure S8-B, r = 0.8496; p<0.0001).

#### Central Asian Factorial Correspondence Analysis

**Figure 4:**
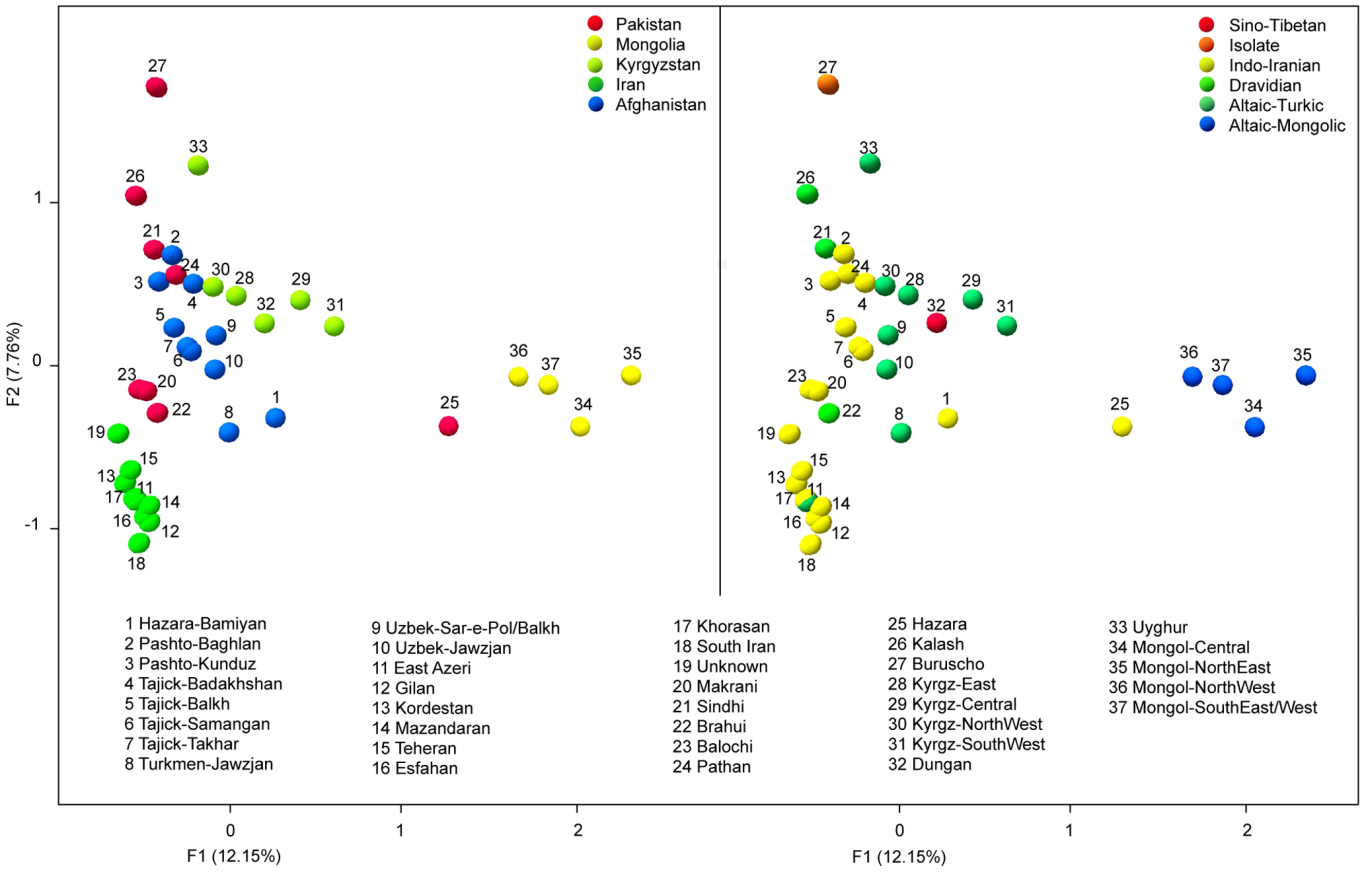
First and second components of the Factorial Correspondence Analysis based on the frequencies of 84 well-defined Y chromosome haplogroups in 37 populations from Afghanistan, Iran, Kyrgyzstan, Pakistan, and Mongolia. In Figure 4-A, populations are colored according to their language (Altaic and Indo-European speaking populations). Figure 4-B differentiates populations according to their respective country.

We ran a FCA on the populations for which the first two axes addressed 20% of total variance (Figures 4-A and 4-B). Figure 4-A reflects the distribution of populations based on their linguistic affiliation; the first axis separates the Altaic-speaking Mongols and Indo-European Pakistani Hazara from the other populations with an introgression of the Altaic-Turkic into the Indo-Iranian speakers. Note that the Indo-European-speaking Hazara from Pakistan and Afghanistan lie within the Altaic cluster. Figure 4-B reflects the distribution of populations based on their geographic affiliation. This representation pinpoints a strong geographic structure (confirmed with AMOVA, see below) in which populations from each country cluster independently with various degrees of homogeneity. Afghan populations are placed in-between the Kyrgyz, Iranian and Pakistani populations suggesting a genetic influence across these parts of East Asia.

FC plots in Figures S9 show 34 Y-chromosome haplogroup frequencies from our Central Asian database (see Table S4) and from the samples in this study (Afghanistan, Iran, Mongolia, Pakistan, Kyrgyzstan). Language affiliation showed that Altaic-speaking populations stretched from peripheral Tungusic, and Mongolic to Turkic, which merge with Dravidian and Indo-European groups. The Indo-European-speaking populations were more spread out, Indo-Aryan and Balto-Slavic were in tight formation, whereas Indo-Iranian speakers were the most dispersed. When geographic affiliation was considered, populations respectively from East, North, South and West Eurasia and from Caucasus displayed well-defined clusters. On the contrary, Central Asian populations were more scattered and at the junction of the five regional clusters.

#### Median Joining Networks

Median Joining Networks were performed on specific lineages chosen for their correlation with geography or for the large size of the sample. Figure S10 displays the Median Joining Networks performed on C3b2b1-M401, J2a1-Page55 and R1a1a-M198. The C3b2b1-M401 network shows few haplotypes, correlated to a signal of expansion. On the contrary, J2a1-Page55 and R1a1a-M198 networks display high heterogeneity both in haplotype and the population sharing these haplotypes, with the exception of some populations such as Hazara (J2a1-Page55) or Pashtun (R1a1a-M198).

#### Spatial correlation of Y-chromosome data

The Mantel test showed significant rank correlation between genetic and geographic distances (rho = 0.332, p = 0.0005). Haplogroup C3-M401 correlated positively with latitude and longitude, whereas J2a1-Page55 correlated negatively. Haplogroup R1a1a-M198 showed no correlation with either latitude or longitude (Table S6).

*AMOVA*

We performed an AMOVA analysis of Y haplogroup frequencies in our 37 populations by comparing language families (Indo-European, Altaic, Dravidian and Sino-Tibetan) and geography (Iran, Pakistan, Afghanistan, Kyrgyzstan and Mongolia). Language grouping was not significant (Fct = −1.65%) whereas geography was significant (Fct = 7.63%; p<0.001).

## Discussion

Central Asia, defined as the region containing Kazakhstan, Uzbekistan, Turkmenistan, Kyrgyzstan, Tajikistan, Afghanistan and the northern part of Pakistan, has gathered a growing and ongoing interest from archaeologists and anthropologists. Retracing the main historical events in the gene pool of the present Afghan populations has been strongly restricted, because of sampling work in this country being inadvised, with the exceptions of recent Y chromosome studies [30]–[32]. Herein, we contribute to fill this gap by providing a detailed genetic picture of the five main ethnic groups inhabiting the mountainous region of the Hindu Kush. Autosomal, mtDNA and Y-chromosome data (including 6 new Y-SNPs) was enriched with 672 original male samples from Iran, Kyrgyzstan, Mongolia and Pakistan and three exhaustive databases from published work. Given the uncertainties associated with Y-STR mutation rates [73] together with the onset of recent estimations of the Time to Most Recent Common Ancestor (TMRCA) of the various branching events in SNP based Y phylogenies using ‘complete’ Y sequences [74]–[76], in prudence, we choose not to estimate expansion times based on Y-STR diversities. The autosomal and haploid genetic pictures of Central Asians were then revised in the light of this original data from Afghanistan.

### Refinement of Y-chromosome haplogroup C phylogeography

We confirmed that the Hazara showed a high degree of East Asian admixture for autosomal and both haploid loci; in accordance with previous reports using genome-wide genotyping data sets [72] and complementary autosomal markers like ADH1B*47His allele [70] or EDAR*370A allele [71]. Despite profound linguistic differences, Hazara and Uygurs were also close, thus confirming previous observations [77], [78]. Some Y-chromosome lineages, especially haplogroup C3, show evidence for an East Asian origin with subsequent gene flow predominantly towards Central Asia.

Several studies reported C3 Y-chromosome haplogroup in Mongols [79], [80] and other north Eurasian populations [81]–[83]. Haplogroup C3 is the most frequent and widespread subclade. Here we improve the phylogenetic resolution within the Y-chromosome haplogroup C3-PK2 by identifying SNPs describing two bifurcating subclades, C3a-M386 and C3b-M532 that accounted for all C3-PK2 derived chromosomes in our dataset. Another improvement to C3 topology involves new sub-haplogroups within the C3b-M532 component including C3b2b1-M401 that circumscribes the Mongol ‘star cluster’ YSTR haplotype [61]. The amplified C3b2b1-M401 signal found in Afghan Hazara and Mongols as well as in the Kyrgyz shows a correlation with latitude and longitude.

The enhancement of resolution within haplogroup C3 has important implications for future studies. First, it should allow tracking of the Mongol invasions by Genghis Khan and identification of affiliated descendants since the 13th century, as well as detection of possible dispersal of C3 lineages during prehistoric migrations [81], [82], [84]. Secondly, the new improved phylogenetic resolution reported here provides new insights into the diversification of this important sub-clade including the component that was involved in the population of the American continent. Thus, better resolution within haplogroup C3 may help localize candidate Siberian precursors of some native North Americans, since phylogenetic analysis of a single native north American C3b1-P39 derived chromosome indicated that the nearest molecular ancestor was C3b-M532*(xM86,M504,M546). The Native American sample derived for P39 used in determining the phylogenetic relationship was the type specimen from the YCC collection described in the original 2002 nomenclature Genome Research paper. For comparison, the native American haplogroup Q precursor has recently been shown to originate from southern Altai [85], [86].

Our haploid data support the scenario of a limited number of family members accompanying Mongol soldiers on foreign expeditions. Family accompaniment was probably subject to further restriction when permanent occupation with subsequent colonization was planned, since these operations required full-scale nomadic life with strict military discipline. Under these circumstances, mixing with the local population was probably extensive. This hypothesis is also supported by the fact that within one century after occupying Southeastern Europe, the Mongols were already speaking Kypchak Turkic. Similarly, the absence of East Asian ancestry components in the classical Persian heartland, clearly shows that political and military control by Genghis Khan and his sons had limited effects on the genetic structure of heavily populated areas like Iran, the Indus Basin or South Caucasus.

### Central Asia as a convergent zone

Central Asia displays very high genetic diversity [32], [41], [72]. This region has been proposed to be the source of waves of migration leading into Europe, the Americas and India [36]. In such a context, the Y-chromosome studies conducted in Afghanistan by Lacau et al. [30], [31] concluded that North Hindu Kush populations display some degree of genetic isolation compared to those in the South, and that Afghan paternal lineages reflect the consequences of pastoralism and recent historical events. However, these studies focused on the Pashtun and our results showed that this ethnic group is not representative of the other Afghan populations. Haber et al. [32] studied 4 ethnic groups from Afghanistan (Hazara, Pashtun, Tajik and Uzbek); they concluded that population structures are highly correlated with ethnicity in Afghanistan.

Our autosomal and haploid data suggested that the Afghan Hindu Kush populations exhibit a blend of components from Europe, the Caucasus, Middle East, East and South Asia. This juxtaposition of autosomal and haploid markers could reflect important male and female influences contributing to the Afghan populations' genetic make-up. Considering autosomal data, all ancestral components displayed a decreasing gradient of their frequencies when approaching Afghanistan. Finding the highest genetic frequencies in a region does not necessarily mean that this region was the original source: it has been shown that geographic distributions can result from various modalities besides natural selection such as geographic barriers, subsequent migrations, replacement, isolation, and the surfing effect [69]. However, the fact that all the ancestral components reach a lower frequency when in Afghanistan supports the model of a convergence of migrations [87], [88]. Concerning haploid markers, the absence of Y-chromosome “star-clusters” such as those observed in the Mongol population, suggests that there have not been any founder events leading to expansions out of Afghanistan; it is noteworthy that the high resolution in this study allowed us to be affirmative on the absence of any “star” haplogroup in the Afghan samples, supporting the hypothesis of a long-range accumulation [46].

Our population data gives continuous genetic cover across Asia independent of language. Whereas the Eurasian main subcontinent components (defined as K = 9 of Admixture Analysis) are consistent with the linguistic spectrum of Macro-Caucasian in the west (Near Eastern agricultural terms) (AC3 & AC6), Indo-Iranian in the north (AC4), Dravidian Brahui in the south (AC7) and Turkic and Mongol in the east (AC8 & AC9); such a linguistic correlation is not to be found in our Afghan samples. In the Hindu Kush region, the autosomal and haploid genetic structure can be explained better by geography than by language or ethnicity; this is in accordance with two recent studies on autosomal STR and blood group from these Afghan samples and compared to published data from surrounding regions [89], [90]. The autosomal STR study conducted on these Afghan samples and compared with STR data from 29 populations from India, Kuwait, Iran, Iraq, Syria, Lebanon, Jordan, Palestine, Yemen, Oman, Saudi Arabia, Pakistan, Bangladesh, Dubaï and Egypt showed that 11 of the 15 STR exhibit a strong and highly significant correlation between genetic and geographic distance [89]. Another study by our team [90] performed on blood groups from these Afghan samples compared to published data from Western Europe, West Asia, South Asia and East Asia, showed that the five Afghan ethnic groups RHCE haplotypic frequencies were at an intermediate level with the neighboring regions. The greater association of genetic patterns with geography rather than with language is also in accordance with a previous study in Pakistan [65] that included some ethnic groups which are also present in Afghanistan. This is, however, in some contrast with the findings of Martines-Cruz et al. [72] and Haber et al. [32] who highlighted a correlation with ethnicity, but could be explained by a less prominent genetic impact of the Turkic speakers who arrived later in the more distant Hindu Kush region. The fact that genetic structure follows geography rather than language in the Afghan Hindu Kush populations may indicate that the current linguistic situation results from sequentially overlapping the languages of the incoming populations. Thus, determination of fundamental genetic affinities in these Afghan populations appears to pre-date the development of present-day languages.

The Inner Asian Mountain Corridor (IAMC) proposed by Frachetti [2] provides a scenario that underlines the common hunter-gatherer background, followed by much more extensive interactions due to inter-regional pastoralism from c. 3000 BC, leading to a common substrate which then extended to neighboring groups. This would have led to the significant grouping due to geography, where the mountains exert more influence, instead of due to language. This interpretation of genetic structure is also consistent with the historical and genetic data of the western side of the Hindu Kush. The expected effect of the historically attested, large Iranian influx in western and southern Central Asia would be homogenization of genetic patterns among populations that are nowadays linguistically unrelated such as the Tajik, Pashtun, Turkmen and Uzbek. Archeologists have uncovered evidence of several epipaleolithic hunter-gatherer sites in northwestern Iran and identified the Zagros Mountains as the likely origin of caprine domestication that subsequently spread into Iran, Turkmenistan and Pakistan during the Neolithic period [44], [45], [91]. The decreasing frequency of the J2a1-Page55 haplogroup toward the east (negative correlation with latitude and longitude) might indicate that epipaleolithic and Neolithic migrations from Iran to Pakistan and Afghanistan may have affected several non-Indo-European languages in the region. Admixture of Tajik from the Ferghana and Oxus valley with northeastern nomads, the future Kyrgyz, Kazakh, and Uzbek speakers (all Turkic speaking now), was a long process [92]. Estimations based on glottochronology indicated that the split between Indo-Aryan and Indo-Iranian proper took place around 4700 years ago [93]. At that time, Kalasha, a Dardic language (Indo-Aryan branch), broke off from Indo-Iranian which is itself ancestral to Persian, Tajiki, Baluchi, Ossetian, just as it is to Indo-Aryan (Vedic Sanskrit, etc.). Accordingly, the Kalasha-speaking population became a genetic isolate possibly because of drift phenomena. Another possible hypothesis is that a significant Mongol-Siberian ancestry component had not reached Central Asia/the Middle East before that time. Indeed, there are no Altaic components in the ancestral Indo-Iranian language. Since this feature is not displayed to a significant extent by present-day Iranian speakers in Iran (Persians), it can be concluded that there had been no such admixture of Indo-Iranians when Indo-Iranians and Indo-Aryans still formed a single group.

## Conclusion

Although the modern Afghan population is made up of ethnically and linguistically diverse groups, the similarity of the underlying gene pool and its underlying gene flows from West and East Eurasia and from South Asia is consistent with prehistoric post-glacial expansions, such as an eastward migration of humans out of the Fertile Crescent in the early Neolithic period, and the arrival of northern steppe nomads speaking the Indo-Iranian variety of Indo-European languages. Taken together, these events led to the creation of a common genetic substratum that has been veneered with relatively recent cultural and linguistic differences.

## Supporting Information

Figure S1 Admixture analysis from K = 2 to 15. Each individual is represented by a vertically (100%) stacked column of ancestry fractions in the constructed population.
(PDF)

Figure S2 Admixture analysis at K = 7 and K = 9. Each individual is represented by a vertically (100%) stacked column of ancestry fractions in the constructed populations. The Hindu Kush populations are labeled in purple. On the zoomed out panel on the right, language families are color coded.
(PDF)

Figure S3 Correlation of latitude and longitude and AC frequencies defined at K = 9 in the admixture analysis. Triangles and squares respectively depict correlation with latitude and longitude. Black plots indicate significant correlation. Correlation was calculated using the Pearson test.
(PDF)

Figure S4 Pairwise FST distances between Central Asia and neighboring populations, ranging from red (low) to blue (high), based on autosomal data. The populations (data from this study and published data [43], [49]–[53], [94] are divided into regional groups.
(PDF)

Figure S5 Central Asia mt-DNA tree. Hierarchic phylogenetic relationships and frequencies (percentages) of the mitochondrial haplogroups observed in the 516 Afghan samples analyzed in the present study. The mutations are scored relative to the RSRS (2); ! denotes a back mutation to ancestral status. Some of the tips are color coded to reflect the most likely geographical origin (or more prevalent at times), and their overall frequencies reported. WA: West Eurasia, SA: South Asia, EA: East Eurasia.
(XLSX)

Figure S6 Mitochondrial DNA FCA. First and second axes of the Factorial Correspondence Analysis based on 50 lineages examined in five Afghan populations and 214 populations previously reported in published data. Population references are listed in Table S3. **S6-A.** Highlight on the main linguistic phyla (Altaic, Caucasian, Dravidian, Indo-European, Sino-Tibetan, Kartvelian). **S6-B.** Altaic phylum dissection (Turkic, Mongolic, Tungusic). **S6-C.** Indo-European phylum dissection (Armenian, Indo-Aryan, Iranian, Slavic). **S6-D.** Highlight on the main Eurasian regions (East Asia, Siberia, South Asia, Central West Asia, Caucasus, Central Asia). **S6-E.** Coordinates of the different variables.
(PDF)

Figure S7 Central Asia Y-chromosome tree. Hierarchic phylogenetic relationships and frequencies (percentages), haplogroup and haplotype diversity of the 84 paternal haplogroups observed in the 87 Pachtuns, 142 Tajiks, 77 Hazaras, 74 Turkmens and 127 Uzbeks from Afghanistan. The following additional population samples were analyzed at comparable Y-chromosome resolution: 186 samples from Iran, 150 samples from Kyrgyzstan, 160 samples from Mongolia, plus 176 samples from Pakistan (HGDP-CEPH). M89, M429, M522, P326, M526 (in italics) were not genotyped but were included for phylogenetic context. In addition, M356, M93, V68, V257, M293, V42, V92, M426, M253, M205, M340, M378, V88 and SRY1532.2 were typed in the present study, but no derived alleles were observed.
(XLSX)

Figure S8 Y-chromosome haplotype and haplogroup diversities. **S8-A.** Y-chromosome haplotype and haplogroup diversities for each population under study. See Figure 1 for population codes. **S8-B** Correlation of Y-chromosome haplotype and haplogroup diversities among populations under study (Pearson r = 0.8496; p<0.0001).
(DOCX)

Figure S9 Y-chromosome FCA. First and second axes of the Factorial Correspondence Analysis based on 34 pooled lineages examined in 37 Central Asian populations and 187 additional ethnic groups previously reported in published data. Population references are listed in supplementary table S3. **S9-A.** Highlight on the main linguistic phyla (Altaic, Caucasian, Dravidian, Indo-European, Sino-Tibetan, Isolate). **S9-B.** Altaic phylum dissection (Turkic, Mongolic, Tungusic). **S9-C.** Indo-European phylum dissection (Armenian, Indo-Aryan, Iranian, Slavic). **S9-D.** Highlight on the main Eurasian regions (East Asia, Siberia, South Asia, Central West Asia, Caucasus, Central Asia). **S9-E.** Y-chromosome tree displaying the consensus lineages used for database construction. **S9-F.** Coordinates of the different variables.
(PDF)

Figure S10 Median-joining networks of Y STR with haplogroups C3b2b1-M401, J2a1-Page55 and R1a1a-M198.
(PPT)

Table S1 Description of Afghan, Mongolian, Kyrgyz and Iranian samples and HGDP-CEPH samples from Pakistan included in the study.
(DOC)

Table S2 List of the samples used for the autosomal analyses: Groups of population, Number of individuals (n), Country/Region of the population and Reference (source).
(XLS)

Table S3 Description of new Y-chromosome binary markers.
(DOC)

Table S4 References used for the mtDNA and the Y-chromosome database.
(DOCX)

Table S5 Y-Chromosome STR profile for each individual in populations from Afghanistan, Iran, Pakistan (CEPH), Mongolia, Kyrgyzstan.
(XLS)

Table S6 Spearman correlation between frequencies of C-M401, J-Page55, R-M17 and Latitude/Longitude of 37 populations.
(DOCX)
