# Supplementary figures and images for: Afghan Hindu Kush: Where Eurasian Sub-Continent Gene Flows Converge
Source: PLoS One. 2013 Oct 18;8(10):e76748. doi: 10.1371/journal.pone.0076748 (PMC3799995; doi:10.1371/journal.pone.0076748)

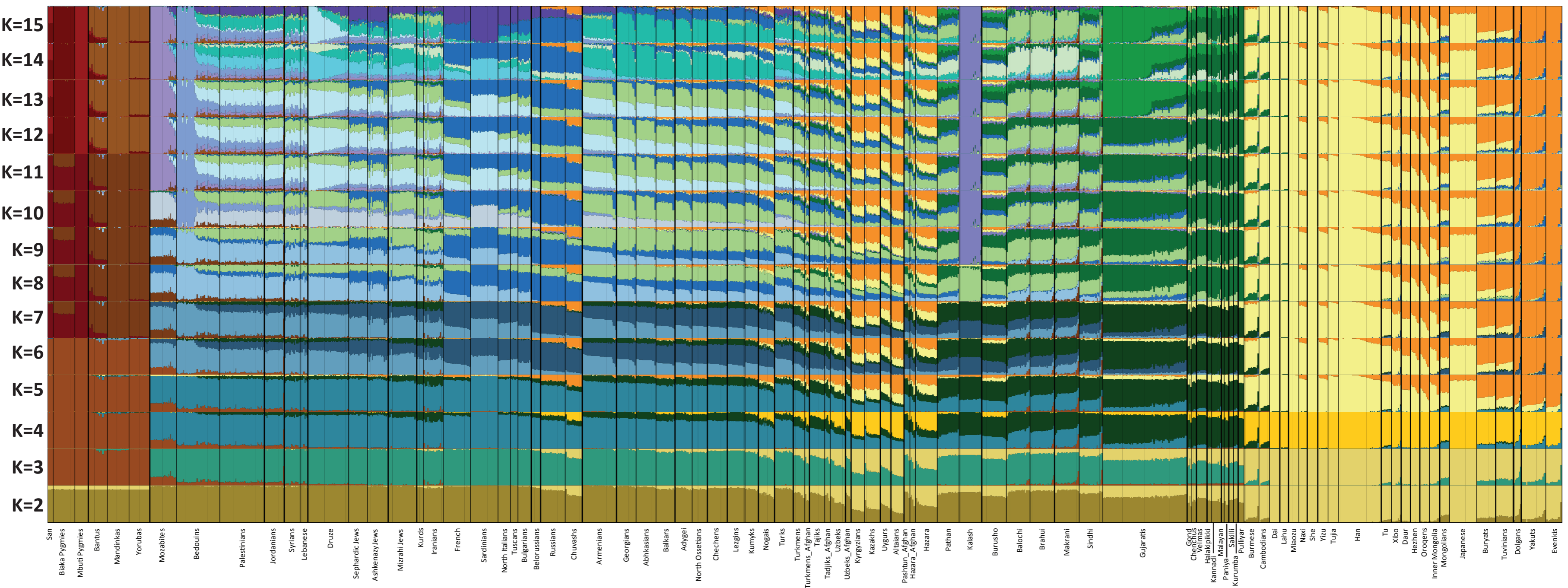

Supplement: Figure S1 — Admixture analysis from K = 2 to 15. Each individual is represented by a vertically (100%) stacked column of ancestry fractions in the constructed population. (PDF) [file pone.0076748.s001.pdf]

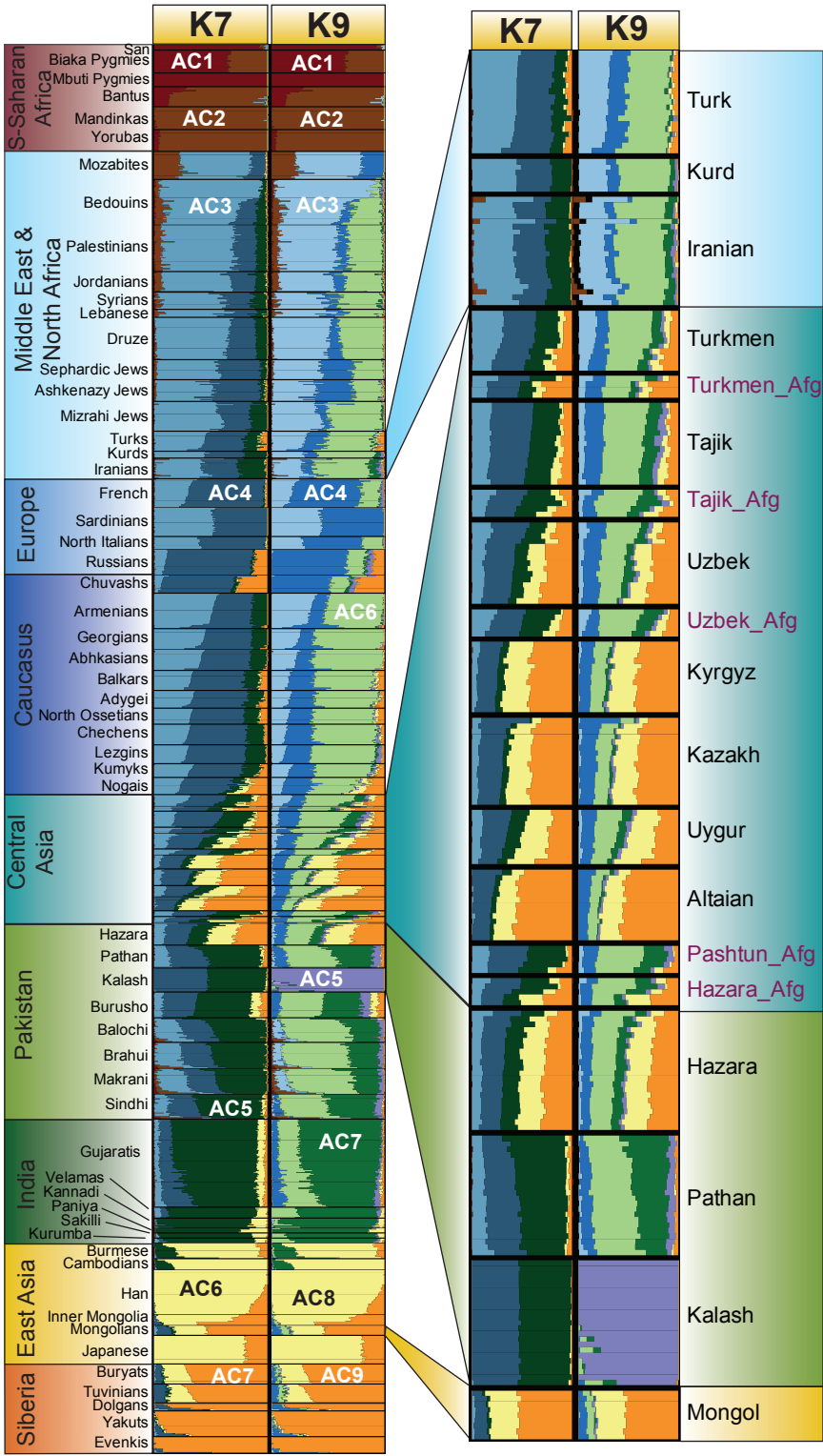

Supplement: Figure S2 — Admixture analysis at K = 7 and K = 9. Each individual is represented by a vertically (100%) stacked column of ancestry fractions in the constructed populations. The Hindu Kush populations are labeled in purple. On the zoomed out panel on the right, language families are color coded. (PDF) [file pone.0076748.s002.pdf]

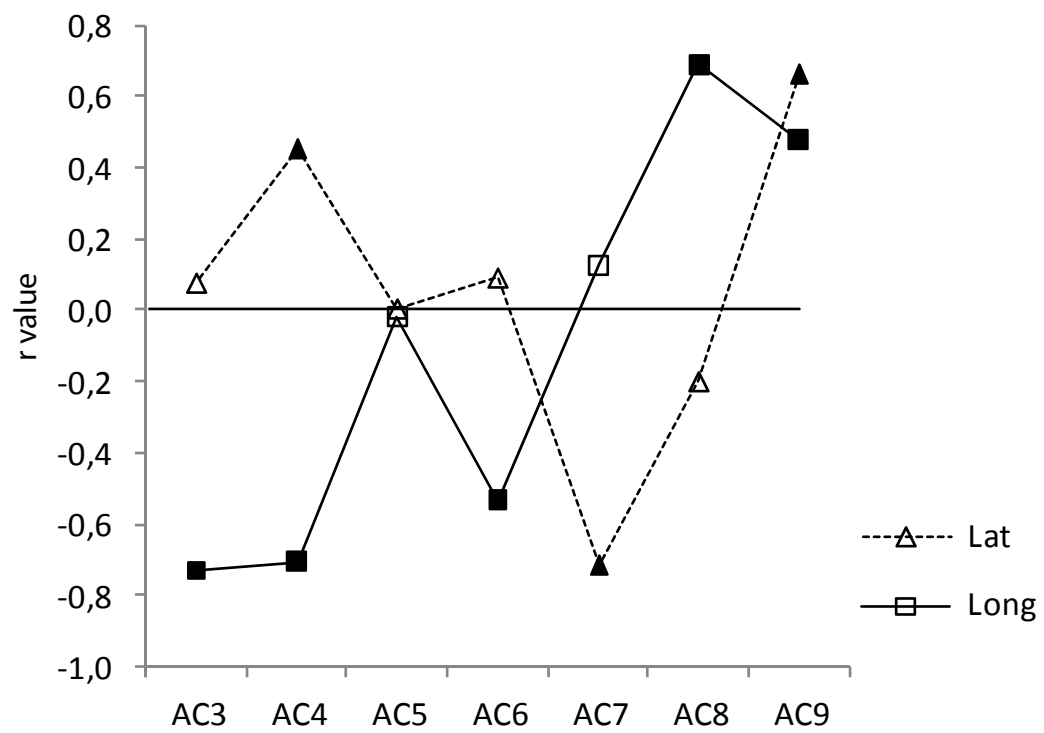

Supplement: Figure S3 — Correlation of latitude and longitude and AC frequencies defined at K = 9 in the admixture analysis. Triangles and squares respectively depict correlation with latitude and longitude. Black plots indicate significant correlation. Correlation was calculated using the Pearson test. (PDF) [file pone.0076748.s003.pdf]

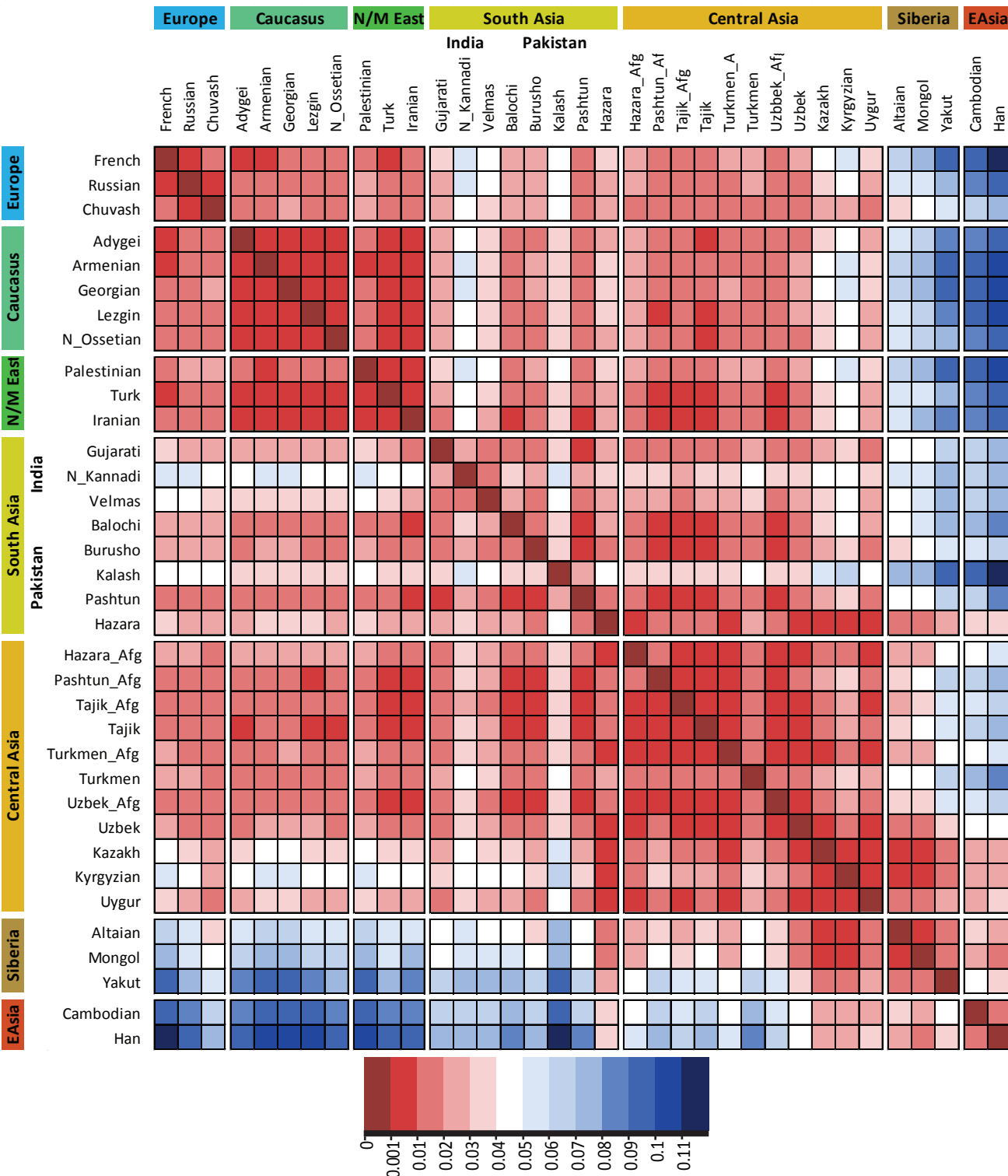

Supplement: Figure S4 — Pairwise FST distances between Central Asia and neighboring populations, ranging from red (low) to blue (high), based on autosomal data. The populations (data from this study and published data [43], [49]–[53], [94] are divided into regional groups. (PDF) [file pone.0076748.s004.pdf]

Factorial Correspondence Analysis - mtDNA

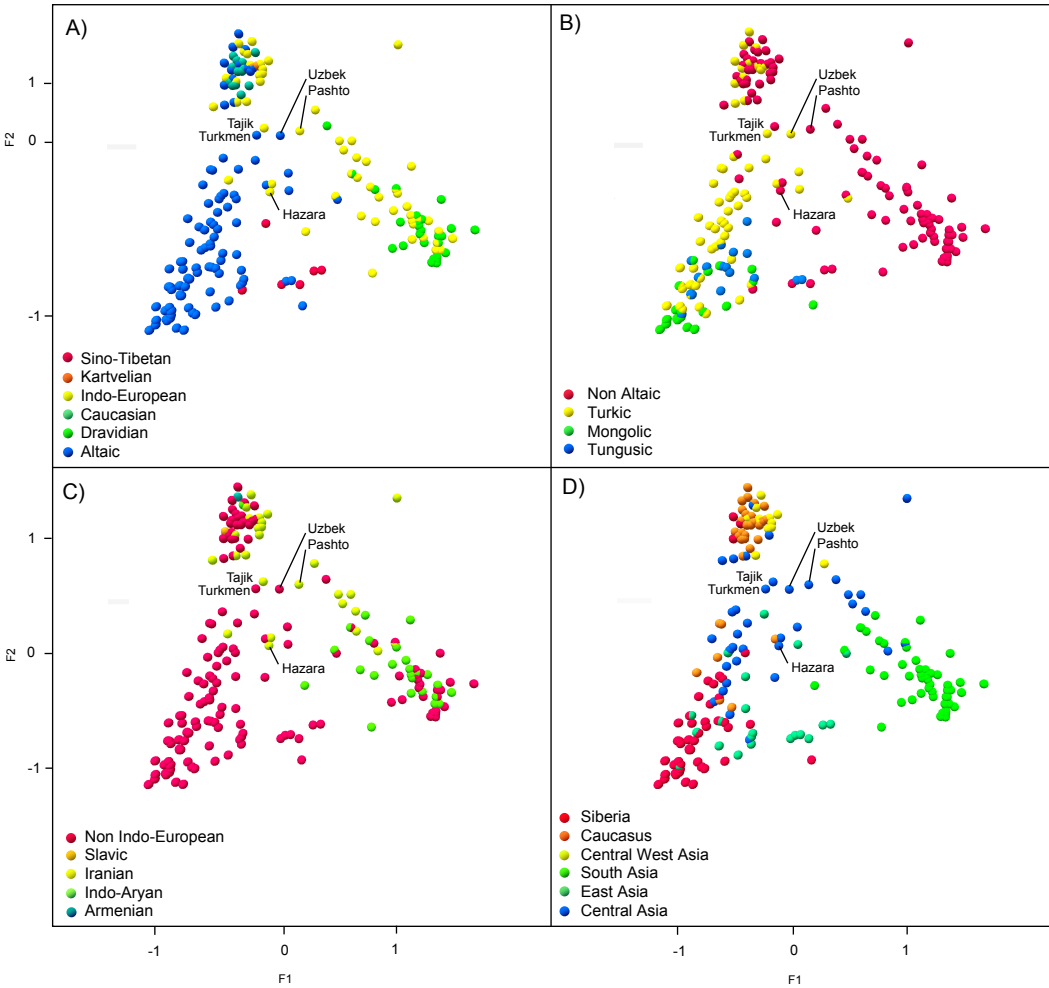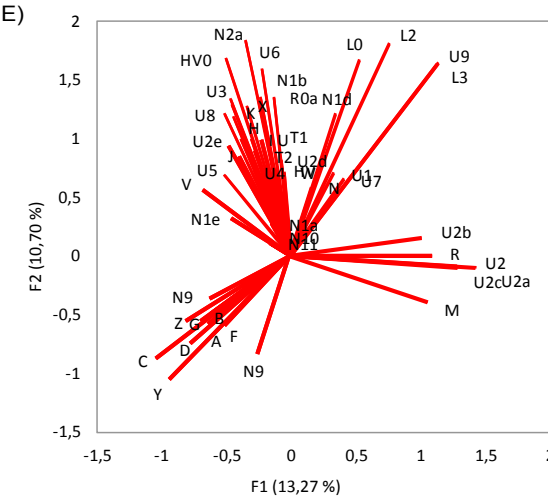

Supplement: Figure S6 — Mitochondrial DNA FCA. First and second axes of the Factorial Correspondence Analysis based on 50 lineages examined in five Afghan populations and 214 populations previously reported in published data. Population references are listed in Table S3. S6-A. Highlight on the main linguistic phyla (Altaic, Caucasian, Dravidian, Indo-European, Sino-Tibetan, Kartvelian). S6-B. Altaic phylum dissection (Turkic, Mongolic, Tungusic). S6-C. Indo-European phylum dissection (Armenian, Indo-Aryan, Iranian, Slavic). S6-D. Highlight on the main Eurasian regions (East Asia, Siberia, South Asia, Central West Asia, Caucasus, Central Asia). S6-E. Coordinates of the different variables. (PDF) [file pone.0076748.s006.pdf]

## Factorial Correspondence Analysis - Y-chr. low resolution

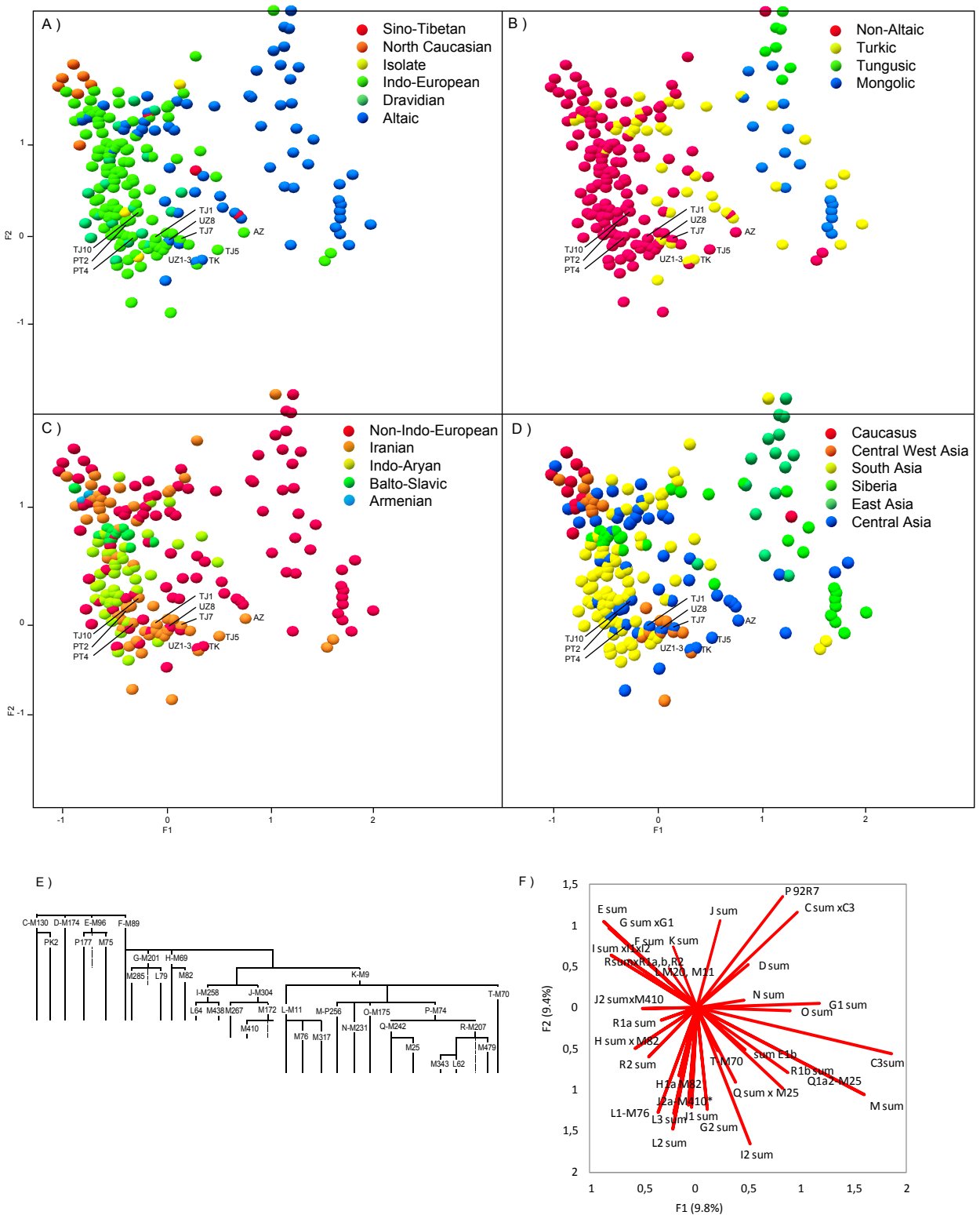

Supplement: Figure S9 — Y-chromosome FCA. First and second axes of the Factorial Correspondence Analysis based on 34 pooled lineages examined in 37 Central Asian populations and 187 additional ethnic groups previously reported in published data. Population references are listed in supplementary table S3. S9-A. Highlight on the main linguistic phyla (Altaic, Caucasian, Dravidian, Indo-European, Sino-Tibetan, Isolate). S9-B. Altaic phylum dissection (Turkic, Mongolic, Tungusic). S9-C. Indo-European phylum dissection (Armenian, Indo-Aryan, Iranian, Slavic). S9-D. Highlight on the main Eurasian regions (East Asia, Siberia, South Asia, Central West Asia, Caucasus, Central Asia). S9-E. Y-chromosome tree displaying the consensus lineages used for database construction. S9-F. Coordinates of the different variables. (PDF) [file pone.0076748.s009.pdf]
