# Supplementary material for: Afghan Hindu Kush: Where Eurasian Sub-Continent Gene Flows Converge
Source: PLoS One. 2013 Oct 18;8(10):e76748. doi: 10.1371/journal.pone.0076748 (PMC3799995; doi:10.1371/journal.pone.0076748)
Supplement: Figure S8 — Y-chromosome haplotype and haplogroup diversities. S8-A. Y-chromosome haplotype and haplogroup diversities for each population under study. See Figure 1 for population codes. S8-B Correlation of Y-chromosome haplotype and haplogroup diversities among populations under study (Pearson r = 0.8496; p<0.0001). (DOCX) [file pone.0076748.s008.docx]

**Figure S8**. Y-chromosome haplotype and haplogroup diversities.

A

B
