# Supplementary material for: Afghan Hindu Kush: Where Eurasian Sub-Continent Gene Flows Converge
Source: PLoS One. 2013 Oct 18;8(10):e76748. doi: 10.1371/journal.pone.0076748 (PMC3799995; doi:10.1371/journal.pone.0076748)
Supplement: Figure S10 — Median-joining networks of Y STR with haplogroups C3b2b1-M401, J2a1-Page55 and R1a1a-M198. (PPT) [file pone.0076748.s010.ppt]

## Slide 1
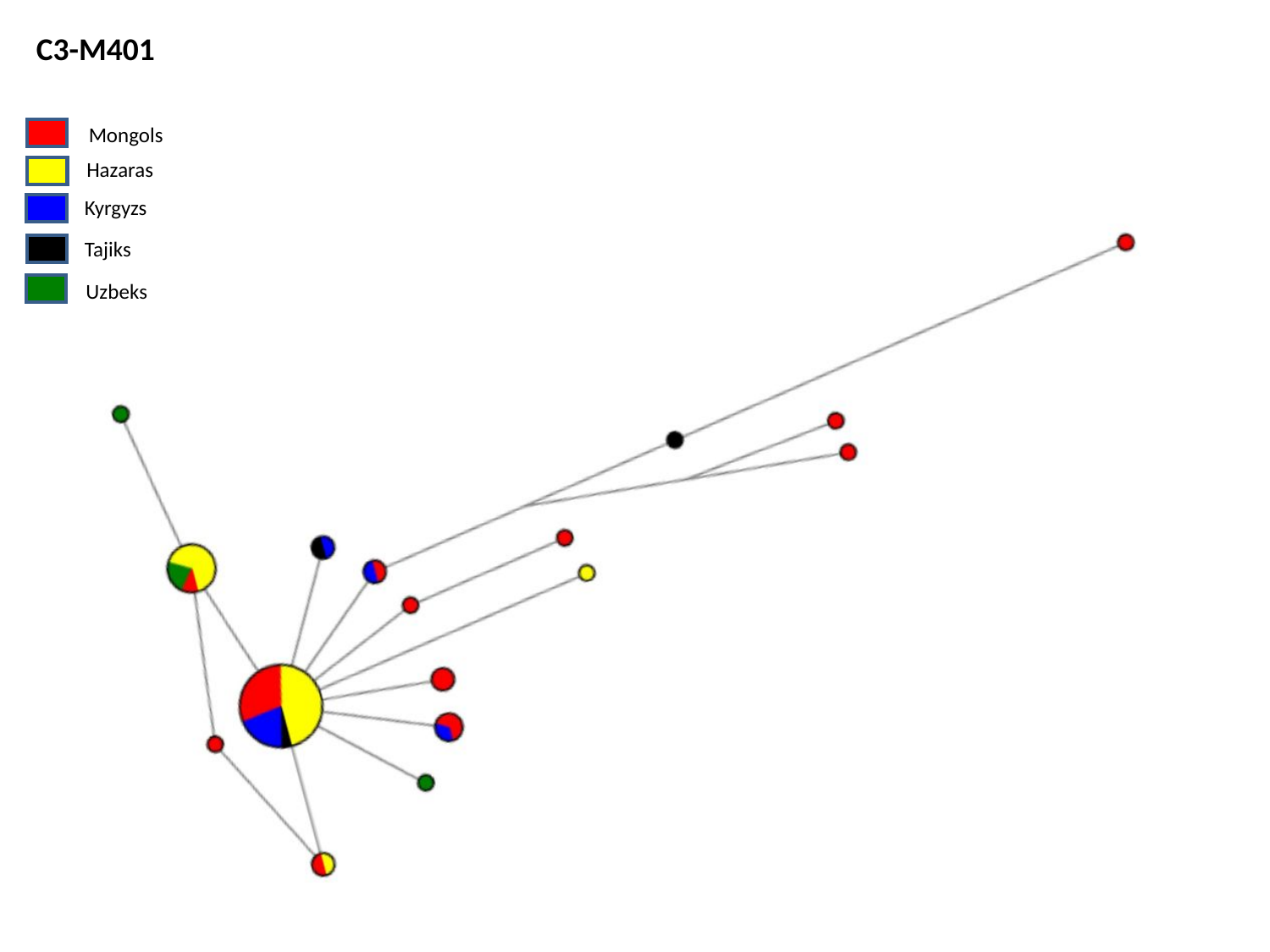

C3-M401
Mongols
Hazaras
Kyrgyzs
Tajiks
Uzbeks

## Slide 2
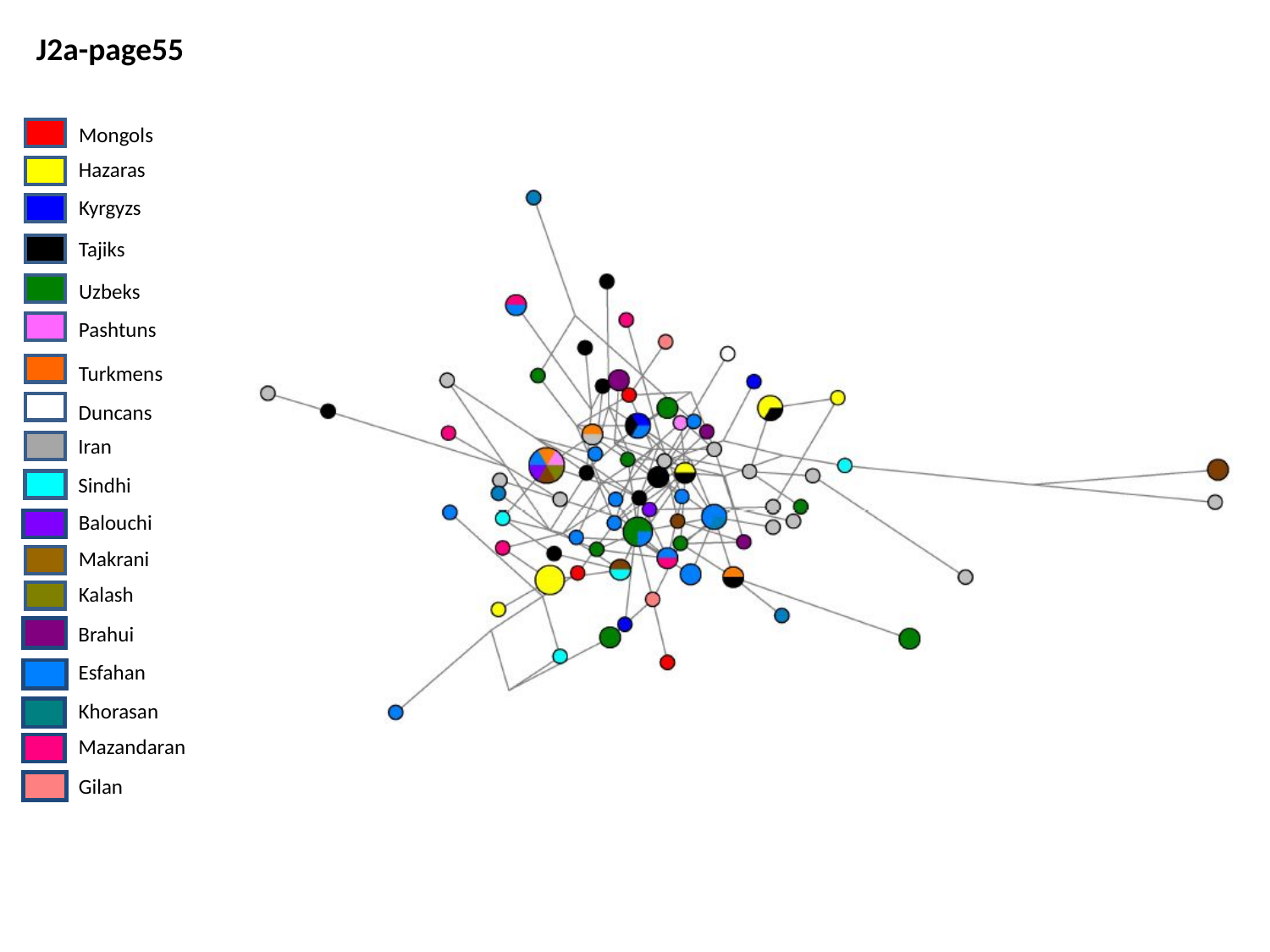

J2a-page55
Mongols
Hazaras
Kyrgyzs
Tajiks
Uzbeks
Pashtuns
Turkmens
Duncans
Iran
Sindhi
Balouchi
Makrani
Kalash
Brahui
Esfahan
Khorasan
Mazandaran
Gilan

## Slide 3
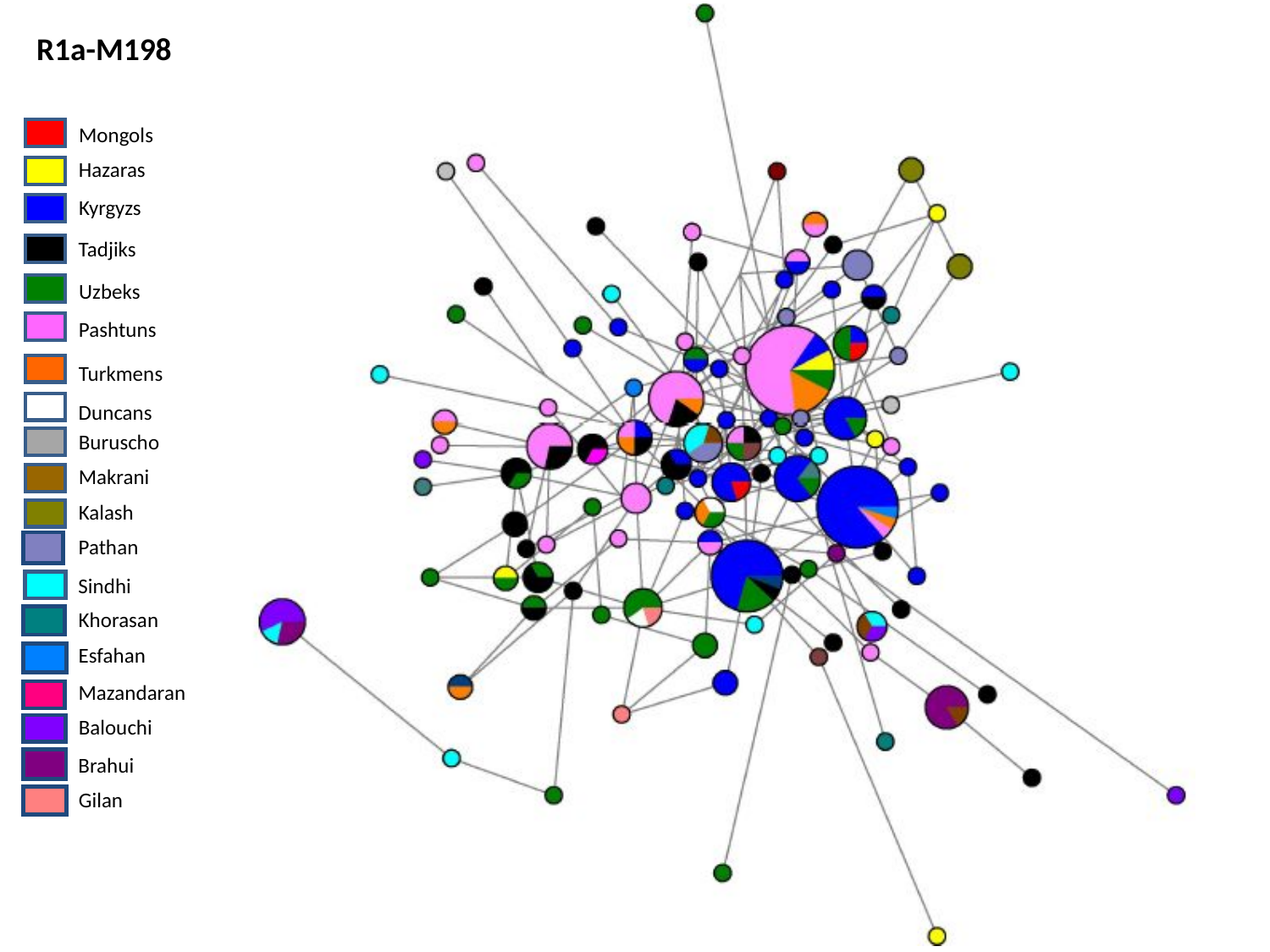

R1a-M198
Mongols
Hazaras
Kyrgyzs
Tadjiks
Uzbeks
Pashtuns
Turkmens
Duncans
Buruscho
Makrani
Kalash
Pathan
Sindhi
Khorasan
Esfahan
Mazandaran
Balouchi
Brahui
Gilan
