# Supplementary material for: Afghan Hindu Kush: Where Eurasian Sub-Continent Gene Flows Converge
Source: PLoS One. 2013 Oct 18;8(10):e76748. doi: 10.1371/journal.pone.0076748 (PMC3799995; doi:10.1371/journal.pone.0076748)
Supplement: Table S1 — Description of Afghan, Mongolian, Kyrgyz and Iranian samples and HGDP-CEPH samples from Pakistan included in the study. (DOC) [file pone.0076748.s011.doc]

Table S1. Description of the 37 country populations under study. Afghan, Mongolian, Kyrgyz and Iranian sample description plus HGDP-CEPH samples from Pakistan included in the study. IE: Indo-European, ALT: Altaic, DRA: Dravidian, IS: Isolate.

| **Populations** | **Codes** | **Country** | **Language** | **N** (Y /mtDNA) | **Latitude** | **Longitude** |
| --- | --- | --- | --- | --- | --- | --- |
| Hazara-Bamiyan | AZ | Afghanistan | IE | 77/78 | 35,61 | 67,21 |
| Pashtun-Baghlan | PT2 |  | IE | 34/35 | 36,29 | 68,29 |
| Pashtun-Kunduz | PT4 |  | IE | 53/52 | 36,96 | 68,21 |
| Tajik-Badakhshan | TJ5 |  | IE | 37 | 37,11 | 70,84 |
| Tajik-Balkh | TJ1 |  | IE | 54/57 | 37,18 | 66,9 |
| Tajik-Samangan | TJ10 |  | IE | 16 | 36,36 | 67,52 |
| Tajik-Takhar | TJ7 |  | IE | 35/37 | 36,7 | 69,45 |
| Turkmen-Jawzjan | TK |  | ALT | 74/75 | 36,6 | 64,74 |
| Uzbek-Sar-e-Pol/Balkh | UZ1-3 |  | ALT | 33/36 | 36,51 | 65,74 |
| Uzbek-Jawzjan | UZ8 |  | ALT | 94/91 | 36,6 | 64,74 |
| East Azeri |  | Iran | ALT | 21 | 38,68 | 47,38 |
| Gilan |  |  | IE | 27 | 36,96 | 49,62 |
| Kordestan |  |  | IE | 25 | 35,09 | 47,23 |
| Mazandaran |  |  | IE | 13 | 36,29 | 52,01 |
| Teheran |  |  | IE | 18 | 32,69 | 54,17 |
| Esfahan |  |  | IE | 42 | 32,77 | 51,55 |
| Khorasan |  |  | IE | 20 | 35,84 | 60,27 |
| South Iran |  |  | IE | 9 | 26,7 | 59,73 |
| Unknown |  |  | IE | 11 | 32,69 | 54,17 |
| Makrani |  | Pakistan  (CEPH) | IE | 20 | 26,03 | 62,5 |
| Sindhi |  |  | IE | 21 | 26,03 | 68,68 |
| Brahui |  |  | DRA | 25 | 29,02 | 62,84 |
| Balochi |  |  | IE | 25 | 30,82 | 66,98 |
| Pathan |  |  | IE | 21 | 32,77 | 70,61 |
| Hazara |  |  | IE | 25 | 33,74 | 70,53 |
| Kalash |  |  | IE | 20 | 36,06 | 71,76 |
| Buruscho |  |  | IS | 20 | 36,36 | 73,85 |
| Kyrgz-East | KG1 | Kyrgyzstan | ALT | 35 | 42,38 | 78,55 |
| Kyrgz-Central | KG2 |  | ALT | 40 | 42,35 | 75,47 |
| Kyrgz-NorthWest | KG3 |  | ALT | 37 | 42,43 | 71,84 |
| Kyrgz-SouthWest | KG4 |  | ALT | 20 | 40,01 | 71,22 |
| Dungan | DG |  | ST | 12 | 42,35 | 75,47 |
| Uyghur | UY |  | ALT | 6 | 42,38 | 78,55 |
| Mongol-Central | MG1 | Mongolia | ALT | 18 | 47,29 | 103,93 |
| Mongol-NorthEast | MG2 |  | ALT | 20 | 48,94 | 114,27 |
| Mongol-NorthWest | MG3 |  | ALT | 97 | 49,24 | 92,98 |
| Mongol-SouthEast-West | MG4-5 |  | ALT | 25 | 45,87 | 113,04 |
