# Supplementary material for: Afghan Hindu Kush: Where Eurasian Sub-Continent Gene Flows Converge
Source: PLoS One. 2013 Oct 18;8(10):e76748. doi: 10.1371/journal.pone.0076748 (PMC3799995; doi:10.1371/journal.pone.0076748)
Supplement: Table S3 — Description of new Y-chromosome binary markers. (DOC) [file pone.0076748.s013.doc]

Table S3 : Description of new Y chromosome binary markers.

| Common Name Marker | YCC nomenclature Haplogroup | Nucleotide change | Amplicon size (bp) reference sequence | Polymorphism position from 5' end of + strand | Restriction enzyme | Position in reference sequence 2009 hg19 Assembly | Position in reference sequence 2006 hg18 Assembly | Primer forward 5'-3' | Primer reverse 5'-3' |
| --- | --- | --- | --- | --- | --- | --- | --- | --- | --- |
| M386 | C3a | C to T | 418 | 206 | Tsp509I | 2738986 | 2798986 | gagatggagtctcactctgttc | ctggcttcctaccacgaatg |
| M532 | C3b | G to T | 407 | 327 | no practical | 19067144 | 17576538 | cttttcctcctaaatggatg | gactaacaacaggctgtgag |
| M533 | D3a1 | C to A | 407 | 317 | AvaII | 19067134 | 17576528 | cttttcctcctaaatggatg | gactaacaacaggctgtgag |
| M504 | C3b2b | T to C | 361 | 179 | BspMI | 21888793 | 20348181 | attttccttattttctaagcagc | cagaataataggagaatttttgg |
| M546 | C3b2b | G to T | 323 | 107 | HphI | 19060966 | 17570360 | gatactttatcaggcttacttc | aaccaaatctctcagaatcg |
| M401 | C3b2b1 | TG insertion | 416 to 418 | 122-123 | no native RFLP | 2745569-70 | 2805569-70 | ctctgcacatgaggagaaaacc | gagatggagtcttgccctatcatc |
