# Supplementary material for: Afghan Hindu Kush: Where Eurasian Sub-Continent Gene Flows Converge
Source: PLoS One. 2013 Oct 18;8(10):e76748. doi: 10.1371/journal.pone.0076748 (PMC3799995; doi:10.1371/journal.pone.0076748)
Supplement: Table S4 — References used for the mtDNA and the Y-chromosome database. (DOCX) [file pone.0076748.s014.docx]

Table S4. References used for the Y chromosome and mtDNA database

| Genetic marker | N° | Citation |
| --- | --- | --- |
| Y-chr. |  |  |
|  | 1. | Al-Zahery N, et al. (2003) Y-chromosome and mtDNA polymorphisms in Iraq, a crossroad of the early human dispersal and of post-Neolithic migrations. *Mol Phylogenet Evol* 28**,** 458-72. |
|  | 2. | Behar DM, et al. (2004) Contrasting patterns of Y chromosome variation in Ashkenazi Jewish and host non-Jewish European populations. *Hum Genet* 114**,** 354-65. |
|  | 3. | Biro AZ, Zalan, A, Volgyi, A & Pamjav, H (2009) A Y-chromosomal comparison of the Madjars (Kazakhstan) and the Magyars (Hungary). *Am J Phys Anthropol* 139**,** 305-10. |
|  | 4. | Bittles AH, Black, ML & Wang, W (2007) Physical anthropology and ethnicity in Asia: the transition from anthropometry to genome-based studies. *J Physiol Anthropol* 26**,** 77-82. |
|  | 5. | Bosch E, et al. (2006) Paternal and maternal lineages in the Balkans show a homogeneous landscape over linguistic barriers, except for the isolated Aromuns. *Ann Hum Genet* 70**,** 459-87. |
|  | 6. | Chen Z, et al. (2011) Brief communication: Y-chromosome haplogroup analysis indicates that Chinese Tuvans share distinctive affinity with Siberian Tuvans. *Am J Phys Anthropol* 144**,** 492-7. |
|  | 7. | Cinnioglu C, et al. (2004) Excavating Y-chromosome haplotype strata in Anatolia. *Hum Genet* 114**,** 127-48. |
|  | 8. | Comas D, et al. (2000) Georgian and kurd mtDNA sequence analysis shows a lack of correlation between languages and female genetic lineages. *Am J Phys Anthropol* 112**,** 5-16. |
|  | 9. | Cordaux R, et al. (2004) Independent origins of Indian caste and tribal paternal lineages. *Curr Biol* 14**,** 231-5. |
|  | 10. | Derenko M, et al. (2006) Contrasting patterns of Y-chromosome variation in South Siberian populations from Baikal and Altai-Sayan regions. *Hum Genet* 118**,** 591-604. |
|  | 11. | Derenko MV, et al. (2002) Polymorphism of Y-chromosome diallelic loci in native populations of Altai-Sayan region. *Rus J Genet* 38**,** 309-314. |
|  | 12. | Dulik MC, Osipova, LP & Schurr, TG (2011) Y-chromosome variation in Altaian Kazakhs reveals a common paternal gene pool for Kazakhs and the influence of Mongolian expansions. *PLoS One* 6**,** e17548. |
|  | 13. | Fechner A, et al. (2008) Boundaries and clines in the West Eurasian Y-chromosome landscape: insights from the European part of Russia. *Am J Phys Anthropol* 137**,** 41-7. |
|  | 14. | Firasat S, et al. (2007) Y-chromosomal evidence for a limited Greek contribution to the Pathan population of Pakistan. *Eur J Hum Genet* 15**,** 121-6. |
|  | 15. | Flores C, et al. (2005) Isolates in a corridor of migrations: a high-resolution analysis of Y-chromosome variation in Jordan. *J Hum Genet* 50**,** 435-41. |
|  | 16. | Gayden T, et al. (2007) The Himalayas as a directional barrier to gene flow. *Am J Hum Genet* 80**,** 884-94. |
|  | 17. | Haber M, et al. (2012) Afghanistan's ethnic groups share a Y-chromosomal heritage structured by historical events. *PLoS One* 7**,** e34288. |
|  | 18. | Hammer MF, et al. (2006) Dual origins of the Japanese: common ground for hunter-gatherer and farmer Y chromosomes. *J Hum Genet* 51**,** 47-58. |
|  | 19. | Hudjashov G, et al. (2007) Revealing the prehistoric settlement of Australia by Y chromosome and mtDNA analysis. *Proc Natl Acad Sci U S A* 104**,** 8726-30. |
|  | 20. | Jin HJ, et al. (2003) Y-chromosomal DNA haplogroups and their implications for the dual origins of the Koreans. *Hum Genet* 114**,** 27-35. |
|  | 21. | Karafet TM, et al. (2002) High levels of Y-chromosome differentiation among native Siberian populations and the genetic signature of a boreal hunter-gatherer way of life. *Hum Biol* 74**,** 761-89. |
|  | 22. | Karafet TM, et al. (1999) Ancestral Asian source(s) of new world Y-chromosome founder haplotypes. *Am J Hum Genet* 64**,** 817-31. |
|  | 23. | Katoh T, et al. (2005) Genetic features of Mongolian ethnic groups revealed by Y-chromosomal analysis. *Gene* 346**,** 63-70. |
|  | 24. | Keyser-Tracqui C, Crubezy, E & Ludes, B (2003) Nuclear and mitochondrial DNA analysis of a 2,000-year-old necropolis in the Egyin Gol Valley of Mongolia. *Am J Hum Genet* 73**,** 247-60. |
|  | 25. | Keyser-Tracqui C, et al. (2006) Population origins in Mongolia: genetic structure analysis of ancient and modern DNA. *Am J Phys Anthropol* 131**,** 272-81. |
|  | 26. | King RJ, et al. (2008) Differential Y-chromosome Anatolian influences on the Greek and Cretan Neolithic. *Ann Hum Genet* 72**,** 205-14. |
|  | 27. | Kivisild T, et al. (2003) The genetic heritage of the earliest settlers persists both in Indian tribal and caste populations. *Am J Hum Genet* 72**,** 313-32. |
|  | 28. | Kumar V, et al. (2007) Y-chromosome evidence suggests a common paternal heritage of Austro-Asiatic populations. *BMC Evol Biol* 7**,** 47. |
|  | 29. | Lacau H, et al. (2012) Afghanistan from a Y-chromosome perspective. *Eur J Hum Genet*. |
|  | 30. | Lell JT, et al. (2002) The dual origin and Siberian affinities of Native American Y chromosomes. *Am J Hum Genet* 70**,** 192-206. |
|  | 31. | Li H, Cho, K, Kidd, JR & Kidd, KK (2009) Genetic landscape of Eurasia and "admixture" in Uyghurs. *Am J Hum Genet* 85**,** 934-7; author reply 937-9. |
|  | 32. | Malyarchuk B, et al. (2010) Phylogeography of the Y-chromosome haplogroup C in northern Eurasia. *Ann Hum Genet* 74**,** 539-46. |
|  | 33. | Marchani EE, et al. (2008) Culture creates genetic structure in the Caucasus: autosomal, mitochondrial, and Y-chromosomal variation in Daghestan. *BMC Genet* 9**,** 47. |
|  | 34. | Martinez L, et al. (2007) Paleolithic Y-haplogroup heritage predominates in a Cretan highland plateau. *Eur J Hum Genet* 15**,** 485-93. |
|  | 35. | Nasidze I, et al. (2004) Mitochondrial DNA and Y-chromosome variation in the caucasus. *Ann Hum Genet* 68**,** 205-21. |
|  | 36. | Nasidze I, et al. (2005) Genetic evidence for the Mongolian ancestry of Kalmyks. *Am J Phys Anthropol* 128**,** 846-54. |
|  | 37. | Nasidze I, et al. (2004) Genetic evidence concerning the origins of South and North Ossetians. *Ann Hum Genet* 68**,** 588-99. |
|  | 38. | Nasidze I, et al. (2005) MtDNA and Y-chromosome variation in Kurdish groups. *Ann Hum Genet* 69**,** 401-12. |
|  | 39. | Nasidze I, et al. (2009) mtDNA and Y-chromosome variation in the Talysh of Iran and Azerbaijan. *Am J Phys Anthropol* 138**,** 82-9. |
|  | 40. | Nasidze I, et al. (2006) Concomitant replacement of language and mtDNA in South Caspian populations of Iran. *Curr Biol* 16**,** 668-73. |
|  | 41. | Nasidze I, et al. (2008) Close genetic relationship between Semitic-speaking and Indo-European-speaking groups in Iran. *Ann Hum Genet* 72**,** 241-52. |
|  | 42. | Nasidze I, Sarkisian, T, Kerimov, A & Stoneking, M (2003) Testing hypotheses of language replacement in the Caucasus: evidence from the Y-chromosome. *Hum Genet* 112**,** 255-61. |
|  | 43. | Pakendorf B, et al. (2006) Investigating the effects of prehistoric migrations in Siberia: genetic variation and the origins of Yakuts. *Hum Genet* 120**,** 334-53. |
|  | 44. | Qamar R, et al. (2002) Y-chromosomal DNA variation in Pakistan. *Am J Hum Genet* 70**,** 1107-24. |
|  | 45. | Quintana-Murci L, et al. (2004) Where west meets east: the complex mtDNA landscape of the southwest and Central Asian corridor. *Am J Hum Genet* 74**,** 827-45. |
|  | 46. | Ramana GV, et al. (2001) Y-chromosome SNP haplotypes suggest evidence of gene flow among caste, tribe, and the migrant Siddi populations of Andhra Pradesh, South India. *Eur J Hum Genet* 9**,** 695-700. |
|  | 47. | Regueiro M, et al. (2006) Iran: tricontinental nexus for Y-chromosome driven migration. *Hum Hered* 61**,** 132-43. |
|  | 48. | Rosser ZH, et al. (2000) Y-chromosomal diversity in Europe is clinal and influenced primarily by geography, rather than by language. *Am J Hum Genet* 67**,** 1526-43. |
|  | 49. | Sahoo S, et al. (2006) A prehistory of Indian Y chromosomes: evaluating demic diffusion scenarios. *Proc Natl Acad Sci U S A* 103**,** 843-8. |
|  | 50. | Semino O, et al. (2000) The genetic legacy of Paleolithic Homo sapiens sapiens in extant Europeans: a Y chromosome perspective. *Science* 290**,** 1155-9. |
|  | 51. | Sengupta S, et al. (2006) Polarity and temporality of high-resolution y-chromosome distributions in India identify both indigenous and exogenous expansions and reveal minor genetic influence of Central Asian pastoralists. *Am J Hum Genet* 78**,** 202-21. |
|  | 52. | Sharma S, et al. (2007) A novel subgroup Q5 of human Y-chromosomal haplogroup Q in India. *BMC Evol Biol* 7**,** 232. |
|  | 53. | Su B, et al. (2000) Polynesian origins: insights from the Y chromosome. *Proc Natl Acad Sci U S A* 97**,** 8225-8. |
|  | 54. | Su B, et al. (1999) Y-Chromosome evidence for a northward migration of modern humans into Eastern Asia during the last Ice Age. *Am J Hum Genet* 65**,** 1718-24. |
|  | 55. | Tajima A, et al. (2004) Genetic origins of the Ainu inferred from combined DNA analyses of maternal and paternal lineages. *J Hum Genet* 49**,** 187-93. |
|  | 56. | Tajima A, et al. (2002) Three major lineages of Asian Y chromosomes: implications for the peopling of east and southeast Asia. *Hum Genet* 110**,** 80-8. |
|  | 57. | Thangaraj K, et al. (2010) The influence of natural barriers in shaping the genetic structure of Maharashtra populations. *PLoS One* 5**,** e15283. |
|  | 58. | Trivedi R, et al. (2007) High Resolution Phylogeographic Map of Y-Chromosomes Reveal the Genetic Signatures of Pleistocene Origin of Indian Populations. *The Anthropologist* 3**,** 393-414. |
|  | 59. | Wells RS, et al. (2001) The Eurasian heartland: a continental perspective on Y-chromosome diversity. *Proc Natl Acad Sci U S A* 98**,** 10244-9. |
|  | 60. | Wen B, et al. (2004) Genetic evidence supports demic diffusion of Han culture. *Nature* 431**,** 302-5. |
|  | 61. | Wen B, et al. (2004) Analyses of genetic structure of Tibeto-Burman populations reveals sex-biased admixture in southern Tibeto-Burmans. *Am J Hum Genet* 74**,** 856-65. |
|  | 62. | Xue Y, et al. (2006) Male demography in East Asia: a north-south contrast in human population expansion times. *Genetics* 172**,** 2431-9. |
|  | 63. | Yao YG, et al. (2004) Different matrilineal contributions to genetic structure of ethnic groups in the silk road region in china. *Mol Biol Evol* 21**,** 2265-80. |
|  | 64. | Zerjal T, et al. (2002) A genetic landscape reshaped by recent events: Y-chromosomal insights into central Asia. *Am J Hum Genet* 71**,** 466-82. |
|  | 65. | Zerjal T, et al. (2003) The genetic legacy of the Mongols. *Am J Hum Genet* 72**,** 717-21. |
|  | 66. | Zhong H, et al. (2011) Extended Y chromosome investigation suggests postglacial migrations of modern humans into East Asia via the northern route. *Mol Biol Evol* 28**,** 717-27. |
|  | 67. | Zhong H, et al. (2010) Global distribution of Y-chromosome haplogroup C reveals the prehistoric migration routes of African exodus and early settlement in East Asia. *J Hum Genet* 55**,** 428-35. |
|  | 68 | Grugni V, Battaglia V, Hooshiar Kashani B, Parolo S, Al-Zahery N, Achilli A, Olivieri A, Gandini F, Houshmand M, Sanati MH, et al. (2012) *PLoS One* 7, e41252. |
| mtDNA |  |  |
|  | 1. | Bamshad MJ, et al. (1998) Female gene flow stratifies Hindu castes. *Nature* 395**,** 651-2. |
|  | 2. | Bermisheva M, Tambets, K, Villems, R & Khusnutdinova, E (2002) [Diversity of mitochondrial DNA haplotypes in ethnic populations of the Volga-Ural region of Russia] article in russian. *Mol Biol (Mosk)* 36**,** 990-1001. |
|  | 3. | Bermisheva MA, et al. (2005) [Analysis of mitochondrial DNA variation in the population of Oroks] article in russian. *Genetika* 41**,** 78-84. |
|  | 4. | Chen F, et al. (2008) Genetic polymorphism of mitochondrial DNA HVS-I and HVS-II of Chinese Tu ethnic minority group. *J Genet Genomics* 35**,** 225-32. |
|  | 5. | Comas D, et al. (2004) Admixture, migrations, and dispersals in Central Asia: evidence from maternal DNA lineages. *Eur J Hum Genet* 12**,** 495-504. |
|  | 6. | Cordaux R, et al. (2003) Mitochondrial DNA analysis reveals diverse histories of tribal populations from India. *Eur J Hum Genet* 11**,** 253-64. |
|  | 7. | Derenko M, et al. (2007) Phylogeographic analysis of mitochondrial DNA in northern Asian populations. *Am J Hum Genet* 81**,** 1025-41. |
|  | 8. | Derenko MV, Malyarchuk, BA, Dambueva, IK & Zakharov, IA (2003) Structure and diversity of the mitochondrial gene pools of south Siberians. *Dokl Biol Sci* 393**,** 557-61. |
|  | 9. | Derenko MV & Shields, GF (1997) [Diversity of mitochondrial DNA nucleotide sequences in three groups of aboriginal inhabitants of Northern Asia] article in russian. *Mol Biol (Mosk)* 31**,** 784-9. |
|  | 10. | Gibert M, et al. (2010) mtDNA variation in the Buryat population of the Barguzin Valley: New insights into the micro-evolutionary history of the Baikal area. *Ann Hum Biol* 37**,** 501-23. |
|  | 11. | Irwin JA, et al. (2010) The mtDNA composition of Uzbekistan: a microcosm of Central Asian patterns. *Int J Legal Med* 124**,** 195-204. |
|  | 12. | Jin HJ, Kim, KC & Kim, W (2010) Genetic diversity of two haploid markers in the Udegey population from southeastern Siberia. *Am J Phys Anthropol* 142**,** 303-13. |
|  | 13. | Jin HJ, Tyler-Smith, C & Kim, W (2009) The peopling of Korea revealed by analyses of mitochondrial DNA and Y-chromosomal markers. *PLoS One* 4**,** e4210. |
|  | 14. | Kivisild T, et al. (1999) Deep common ancestry of indian and western-Eurasian mitochondrial DNA lineages. *Curr Biol* 9**,** 1331-4. |
|  | 15. | Kivisild T, et al. (2003) The genetic heritage of the earliest settlers persists both in Indian tribal and caste populations. *Am J Hum Genet* 72**,** 313-32. |
|  | 16. | Kolman CJ, Sambuughin, N & Bermingham, E (1996) Mitochondrial DNA analysis of Mongolian populations and implications for the origin of New World founders. *Genetics* 142**,** 1321-34. |
|  | 17. | Kong QP, et al. (2003) Mitochondrial DNA control region and cytochrome b sequence variation in the genus Mystacoleucus Gunther (Pisces: Cyprinidae: Barbinae) from China. *Biochem Genet* 41**,** 305-13. |
|  | 18. | Metspalu M, et al. (2004) Most of the extant mtDNA boundaries in south and southwest Asia were likely shaped during the initial settlement of Eurasia by anatomically modern humans. *BMC Genet* 5**,** 26. |
|  | 19. | Mountain JL, et al. (1995) Demographic history of India and mtDNA-sequence diversity. *Am J Hum Genet* 56**,** 979-92. |
|  | 20. | Pakendorf B, Novgorodov, IN, Osakovskij, VL & Stoneking, M (2007) Mating patterns amongst Siberian reindeer herders: inferences from mtDNA and Y-chromosomal analyses. *Am J Phys Anthropol* 133**,** 1013-27. |
|  | 21. | Puzyrev VP, et al. (2003) [MtDNA and Y-chromosome lineages in the Yakut population] article in russian. *Genetika* 39**,** 975-81. |
|  | 22. | Quintana-Murci L, et al. (2004) Where west meets east: the complex mtDNA landscape of the southwest and Central Asian corridor. *Am J Hum Genet* 74**,** 827-45. |
|  | 23. | Quintana-Murci L, et al. (1999) Genetic evidence of an early exit of Homo sapiens sapiens from Africa through eastern Africa. *Nat Genet* 23**,** 437-41. |
|  | 24. | Rakha A, et al. (2011) Forensic and genetic characterization of mtDNA from Pathans of Pakistan. *Int J Legal Med* 125**,** 841-8. |
|  | 25. | Reidla M, et al. (2003) Origin and diffusion of mtDNA haplogroup X. *Am J Hum Genet* 73**,** 1178-90. |
|  | 26. | Richards M, et al. (2000) Tracing European founder lineages in the Near Eastern mtDNA pool. *Am J Hum Genet* 67**,** 1251-76. |
|  | 27. | Roychoudhury S, et al. (2001) Genomic structures and population histories of linguistically distinct tribal groups of India. *Hum Genet* 109**,** 339-50. |
|  | 28. | Starikovskaya EB, et al. (2005) Mitochondrial DNA diversity in indigenous populations of the southern extent of Siberia, and the origins of Native American haplogroups. *Ann Hum Genet* 69**,** 67-89. |
|  | 29. | Wang W, et al. (2003) The origins and genetic structure of three co-resident Chinese Muslim populations: the Salar, Bo'an and Dongxiang. *Hum Genet* 113**,** 244-52. |
|  | 30. | Yao YG, et al. (2002) Phylogeographic differentiation of mitochondrial DNA in Han Chinese. *Am J Hum Genet* 70**,** 635-51. |
|  | 31. | Yao YG, et al. (2004) Different matrilineal contributions to genetic structure of ethnic groups in the silk road region in china. *Mol Biol Evol* 21**,** 2265-80. |
|  | 32. | Yao YG, et al. (2000) Gene admixture in the silk road region of China: evidence from mtDNA and melanocortin 1 receptor polymorphism. *Genes Genet Syst* 75**,** 173-8. |
