# Supplementary material for: Afghan Hindu Kush: Where Eurasian Sub-Continent Gene Flows Converge
Source: PLoS One. 2013 Oct 18;8(10):e76748. doi: 10.1371/journal.pone.0076748 (PMC3799995; doi:10.1371/journal.pone.0076748)
Supplement: Table S6 — Spearman correlation between frequencies of C-M401, J-Page55, R-M17 and Latitude/Longitude of 37 populations. (DOCX) [file pone.0076748.s016.docx]

Table S6. Spearman correlation between frequencies of C-M401, J-Page55, R-M17 and Latitude/Longitude of 37 populations

| **Haplogroup** |  | **Latitude** | **Longitude** |
| --- | --- | --- | --- |
| C-M401 | Correlation Coefficient | 0.466 | 0.537 |
|  | Sig. (2-Tailed) | 0.004 | 0.001 |
|  | N | 37 | 37 |
| J-Page55 | Correlation Coefficient | -0.541 | -0.700 |
|  | Sig. (2-Tailed) | 0.001 | 0.000 |
|  | N | 37 | 37 |
| R-M17 | Correlation Coefficient | -0.037 | 0.084 |
|  | Sig. (2-Tailed) | 0.827 | 0.620 |
|  | N | 37 | 37 |
